# Supplementary material for: Modelling structural determinants of ventilation heterogeneity: A perturbative approach
Source: PLoS One. 2018 Nov 29;13(11):e0208049. doi: 10.1371/journal.pone.0208049 (PMC6264152; doi:10.1371/journal.pone.0208049)
Supplement: S1 Table — (PDF) [file pone.0208049.s001.pdf]

**Parameter**	**Description**	**Values used**
V_{FRC}	Total lung and mouth cavity volume at rest.	3 L
V_D	Total volume of conducting airways.	0.12 L
V_{mouth}	Total volume of mouth cavity.	0.05 L
V_T	Tidal volume.	1 L
τ	Breath time (inhalation or exhalation).	2.5 s
$V_{\text{duct}}/V_{\text{acin}}$	Proportion of acinar gas volume in ducts.	0.2 [47]
λ_{cond}	Airway length scaling in mean-path conducting branches.	0.794 [5,48]
λ_{acin}	Duct length scaling in acinar branches.	0.93 [47,49]
LD_{cond}	Ratio of length to diameter in mean-path conducting branches.	3 [6,50]
LD_{acin}	Ratio of length to diameter in acinar branches.	2.3 [47]
$N^{\text{acin}} + 1$	Number of acinar airway generations.	9 [47]
Φ_j	Density of alveolar sacs.<br>in acinar generations.<br>$j = N_z^{\text{cond}} \ldots N^{\text{tot}}$.	0 if $j < N_z^{\text{cond}}$<br>0.2 if $j = N_z^{\text{cond}}$<br>0.4 if $j = N_z^{\text{cond}} + 1$ [47]<br>0.7 if $j = N_z^{\text{cond}} + 2$<br>1 if $j > N_z^{\text{cond}} + 2$
ϕ	Phenomenological parameter, see eq. (4).	0.5 [12]
C	Dimensionless constant.	1.08 on inspiration<br>0.37 on expiration [22].
K_{lung}	Elasticity of the whole lung.	5×10^5Pa m^{-3} [51,52]
R_{acin}	Cumulative resistance of acini.	2×10^4Pa s m^{-3} [51,53]
R_{UA}	Resistance of the upper airway.	6×10^4 Pa s m^{-3} [51,53]
μ	Air viscosity at 37°C.	1.93×10^{-5} Pa s
$\{d, l\}$<br>Diameters<br>and lengths<br>of proximal<br>bronchi<br>(cm)	Trachea<br>Right Main Bronchus<br>Left Main Bronchus<br>Right Intermediate Bronchus<br>Right Lower Lobar Bronchus<br>Left Lower Lobar Bronchus	{1.6, 10}<br>{1.11, 2.2}<br>{1.2, 5} [6]<br>{0.89, 2.6}<br>{0.64, 0.8}<br>{0.8, 1.1}
