# Supplementary material for: Modelling structural determinants of ventilation heterogeneity: A perturbative approach
Source: PLoS One. 2018 Nov 29;13(11):e0208049. doi: 10.1371/journal.pone.0208049 (PMC6264152; doi:10.1371/journal.pone.0208049)
Supplement: S2 Table — (PDF) [file pone.0208049.s002.pdf]

**Symbol**	**Description**
R_{cond}	Full matrix comprised of conducting airway resistances.
R_{acin}	Diagonal matrix of acinar resistances.
K	Diagonal matrix of acinar elastances.
$\mathbf{V}(t)$, $\mathbf{V}^*$	Vector of acinar unit volumes at time t, and at FRC (with zero flow) respectively.
$P_{\text{pl}}(t), P_{\text{pl0}}$	Applied pleural pressure at time t, and at FRC (with zero flow) respectively.
s_i, S_i	Total inner and outer airway cross-section on edge i of the lung network respectively.
c_i	Inert gas concentration on edge i.
u_i	Air flow velocity on edge i.
D_i, D_0	Effective and molecular diffusion constants respectively.
a_i, l_i	Cross-section and length of the individual airway i respectively.<br>$s_i = N_b a_i$ where N_b is the number of branches represented by edge i in the network.
$\epsilon_i^{(a)}$, $\epsilon_i^{(l)}$, $\epsilon_\alpha^{(K)}$	Non-dimensionalised perturbation to area, length and elastance<br>of airway i or acinus α respectively.
σ_a^2, σ_l^2	Variance of non-dimensionalised perturbations applied to<br>airway areas and lengths respectively.
