# Supplementary figures and images for: Modelling structural determinants of ventilation heterogeneity: A perturbative approach
Source: PLoS One. 2018 Nov 29;13(11):e0208049. doi: 10.1371/journal.pone.0208049 (PMC6264152; doi:10.1371/journal.pone.0208049)

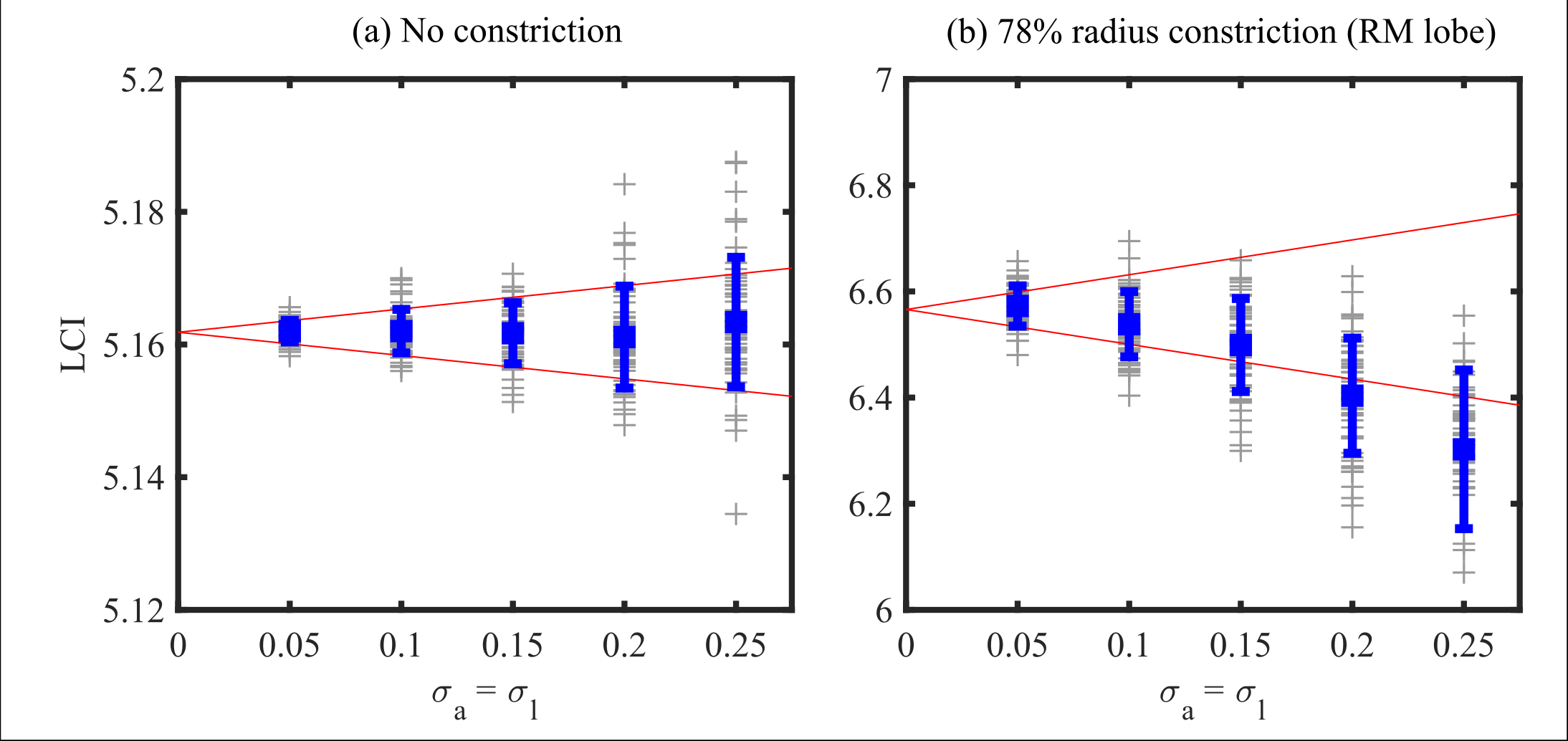

Supplement: S1 Fig — In both cases airway perturbations are assumed independently normally distributed with same coefficient of variation in area and length (σa = σl) and variance in elasticity is not considered σK = 0. Perturbations are only drawn for airways down to and including Strahler order 14, with the remaining generations assumed to be perfectly symmetric (as in model M). For the Monte Carlo prediction, the normal distribution of perturbations is truncated to prevent unphysical behaviour and preserve symmetry such that -1<ϵi(a),ϵi(a)<1. Error bars indicate mean ± one s.d. of model outputs and red lines show the prediction of mean ± one s.d. from model P. Crosses mark the results of individual realisations in the Monte Carlo algorithm. The results are shown for the case when perturbations are applied to (a) the healthy model M, and (b) model M with severe constrictions in the proximal airways of the RM lobe. Good agreement for the predicted variance is observed in both cases up to σa = σl = 0.25, however in the constricted case heterogeneity tends to result in a lower mean LCI, which is not captured by model P. (TIF) [file pone.0208049.s003.tif]

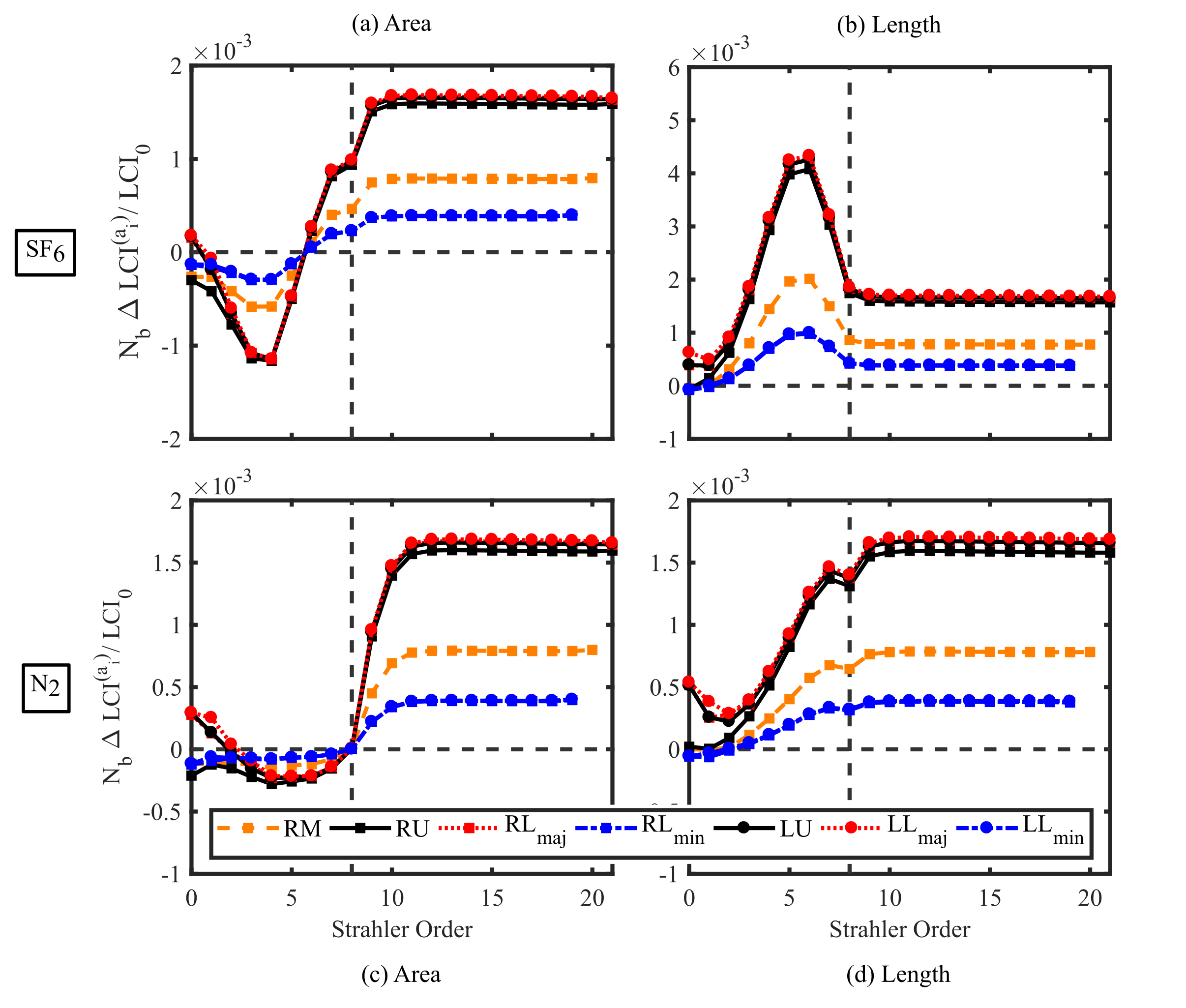

Supplement: S2 Fig — The airway generation is plotted in terms of its Strahler order (i.e. its generation counting up from zero at the bottom of the tree). The vertical dashed line indicate the terminal bronchiole separating the acinar (Strahler orders 0-8) and conducting (>9) generations. (a)-(b) Healthy lung model (no constrictions) using SF6 (molecular diffusivity 0.105cm2 s-1). (c)-(d) Healthy lung model using N2 (molecular diffusivity 0.225cm2 s-1). Coloured symbols distinguish perturbations in the seven lobar regions. The LCI sensitivities in the conducting region (right of the vertical dashed line) are approximately identical for area and length perturbations in both cases, as this is a response to the increase in dead-space volume. For SF6 the sensitivities in the acinar region (left of the vertical dashed line) are inverted for length and area perturbations, most notably around the diffusion front (approximately Strahler order 4). Thus LCI is sensitive to geometry changes that affect diffusion in the acinus when using the less diffusive SF6, but not N2. (TIF) [file pone.0208049.s004.tif]

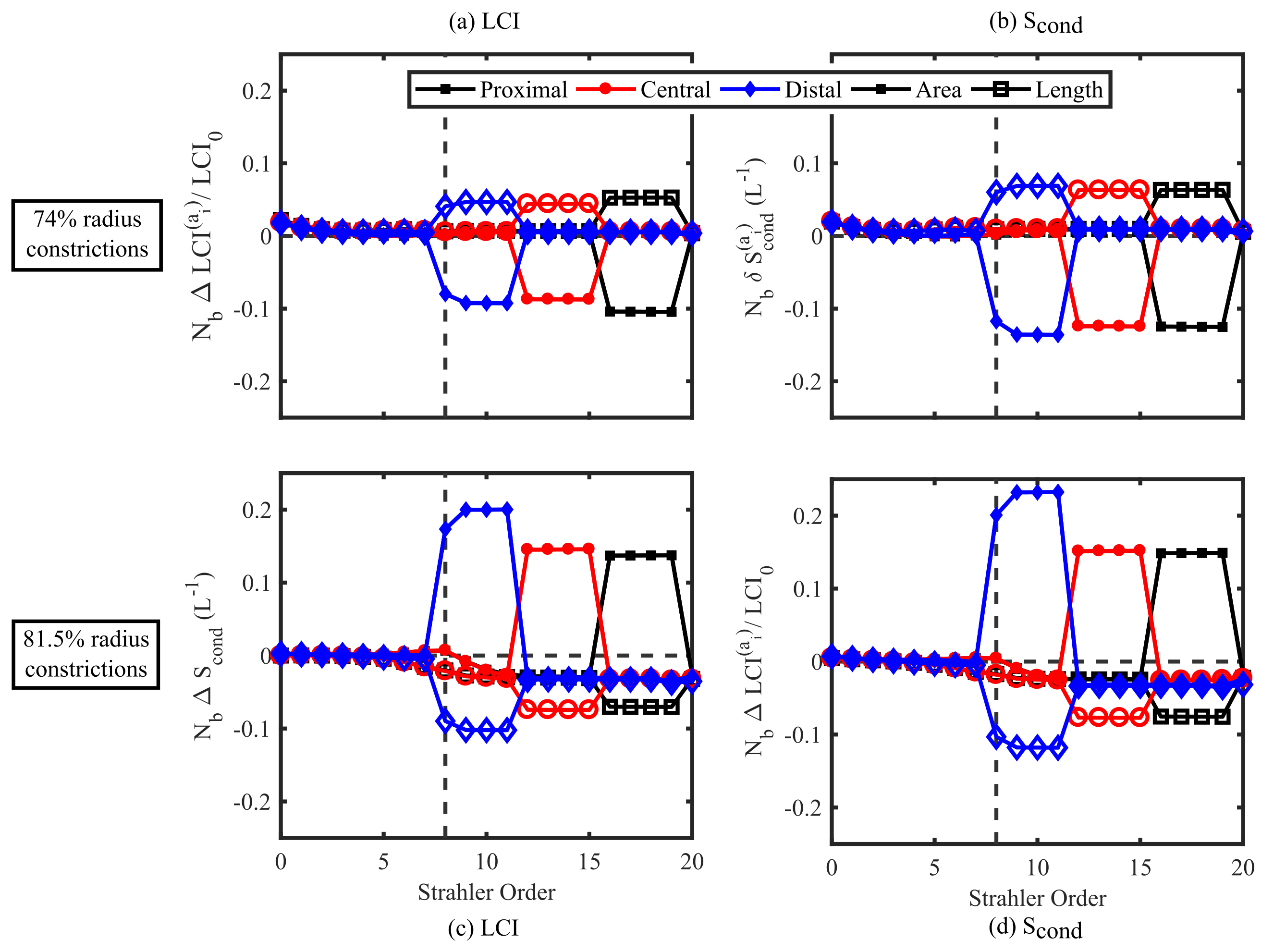

Supplement: S3 Fig — Scaled sensitivities (as in S2 Fig) w.r.t. area (filled markers) and length (open markers) of the airways are shown for the RM lobe only for fractional LCI change and absolute change in Scond. Results were plotted for different depths of constriction: proximal (Strahler orders 16-19, black squares), central (Strahler orders 12-15, red circles) and distal (Strahler orders 8-11, blue diamonds). The sensitivities are scaled by the number of airways in the corresponding Strahler order of the RM lobe. The scaled sensitivities are much larger in the constricted airways, as the response is most sensitive to their resistance (note the difference in scale to S2 Fig). Since airway resistance scales as length/area2, the area sensitivities are approximately a factor −2 of the length sensitivities. The sign of the sensitivities changes between the two constriction strengths because they lie either side of the maximum values of LCI and Scond in Fig 4(a) and 4(b). (TIF) [file pone.0208049.s005.tif]
