# Supplementary material for: Modelling structural determinants of ventilation heterogeneity: A perturbative approach
Source: PLoS One. 2018 Nov 29;13(11):e0208049. doi: 10.1371/journal.pone.0208049 (PMC6264152; doi:10.1371/journal.pone.0208049)
Supplement: S1 File — (PDF) [file pone.0208049.s008.pdf]

Supplementary Text: Modelling structural determinants of ventilation heterogeneity: a perturbative approach.

Carl A. Whitfield*[1,2], Alex Horsley[1], and Oliver E. Jensen[2]

[1]Division of Infection, Immunity and Respiratory Medicine, University of Manchester, Southmoor Road, Manchester, UK, M23 9LT

[2]School of Mathematics, University of Manchester, Oxford Road, Manchester, M13 9PL

September 26, 2018

1 Lung network model

In both models (M and P) the airway network (labelled $\mathcal{L}$) terminates in seven sub-networks that we label $\mathcal{T}_z$ for $z \in \{\text{RU, RM, RL}_{\text{min}}, \text{RL}_{\text{maj}}, \text{LU, LL}_{\text{min}}, \text{LL}_{\text{maj}}\}$ representing the lobes, where the lower lobes are split into major and minor sub-trees (see Fig 1 in main text). The networks $\mathcal{T}_z \in \mathcal{L}$ are each modelled as symmetrically-branching dyadic (SBD) trees, meaning that all of the paths in each network have the same number of bifurcations. The networks $\mathcal{T}_z$ are connected by sets of edges and vertices ($\mathcal{E}^{\text{PA}}, \mathcal{V}^{\text{PA}}$ respectively) representing the proximal airways, given by the data of [1] (as listed in S1 Table). Thus the whole lung network $\mathcal{L} = \mathcal{T}_z \in \mathcal{L}, \mathcal{E}^{\text{PA}}, \mathcal{V}^{\text{PA}}$. The regions $\mathcal{T}_z$ represent different proportions of the lung volume, and are connected asymmetrically by $\mathcal{E}^{\text{PA}}, \mathcal{V}^{\text{PA}}$ and so the network has inter-regional asymmetry in the branching structure, but not intra-regional asymmetry.

1.1 Symmetrically-branching dyadic tree notation

Each SBD airway tree (see figure SF1) is represented as a one-dimensional network $\mathcal{T}_z = \{\mathcal{V}_z, \mathcal{E}_z\}$ of vertices $\mathcal{V}_z = \left\{\mathcal{V}_z^{\text{cond}}, \mathcal{V}_z^{\text{acin}}\right\}$ such that $\mathcal{V}_z^{\text{cond}} = \left\{v_{z,o}, \left\{v_{z,j,k} \mid 0 \le j < N_z^{\text{cond}}, 0 \le k < 2^j\right\}\right\}$ is the subset of vertices in the N_z^{cond} generations of conducting airways in the tree where $v_{z,o}$ is the root vertex. The subset $\mathcal{V}_z^{\text{acin}} = \left\{v_{z,j,k} \mid 0 \le j - N_z^{\text{cond}} < N^{\text{acin}}, 0 \le k < 2^j\right\}$ is the subset of vertices within the N^{acin} generations of acinar airways (taken to be the same in all lung regions). Subsets of edges are similarly identified such that $\mathcal{E}_z = \left\{\mathcal{E}_z^{\text{cond}}, \mathcal{E}_z^{\text{acin}}\right\}$ where $\mathcal{E}_z^{\text{cond}} = \left\{e_{z,0,0} = \{v_{z,o}, v_{z,0,0}\}, \left\{e_{z,j,k} = \left\{v_{z,j-1,\lfloor k/2 \rfloor}, v_{z,j,k}\right\} \mid v_{z,j,k} \in \mathcal{V}_z^{\text{cond}} \setminus v_{z,o}\right\}\right\}$. The subset of acinar airways is $\mathcal{E}_z^{\text{acin}} = \left\{e_{z,j,k} = \left\{v_{z,j-1,\lfloor k/2 \rfloor}, v_{z,j,k}\right\} \mid v_{z,j,k} \in \mathcal{V}_z \setminus \mathcal{V}_z^{\text{cond}}\right\}$. Each vertex in the set of terminal vertices $\Gamma_z \equiv \mathcal{V}_z^{\text{cond}} \cap \mathcal{V}_z^{\text{acin}}$ is connected to the root vertex by a unique (uni-directional) path denoted $\mathcal{P}_{z,o,\alpha} = \left\{e_{z,j,k} \mid j = 0 \ldots N_z^{\text{cond}}, k = \left\lfloor \alpha 2^{j-N_z^{\text{cond}}} \right\rfloor\right\}$. Sub-trees are defined as subsets of vertices and edges in $\mathcal{T}_z$ that form complete trees terminating at the base of $\mathcal{T}_z$ such that $\mathcal{S}_{z,j,k} = \left\{v_{z,j-1,\lfloor k/2 \rfloor}, \left\{v_{z,\tilde{j},\tilde{k}}, e_{z,\tilde{j},\tilde{k}} \mid j \le \tilde{j} < N_z^{\text{cond}} + N^{\text{acin}}, k2^j \le \tilde{k} < (k+1)2^j\right\}\right\}$.

To compute the distribution of fluxes on the tree network (as outlined in section 1.2) we consider each acinus as a single three-dimensional volume consisting of numerous alveolar ducts and sacs, that

*carl.whitfield@physics.org

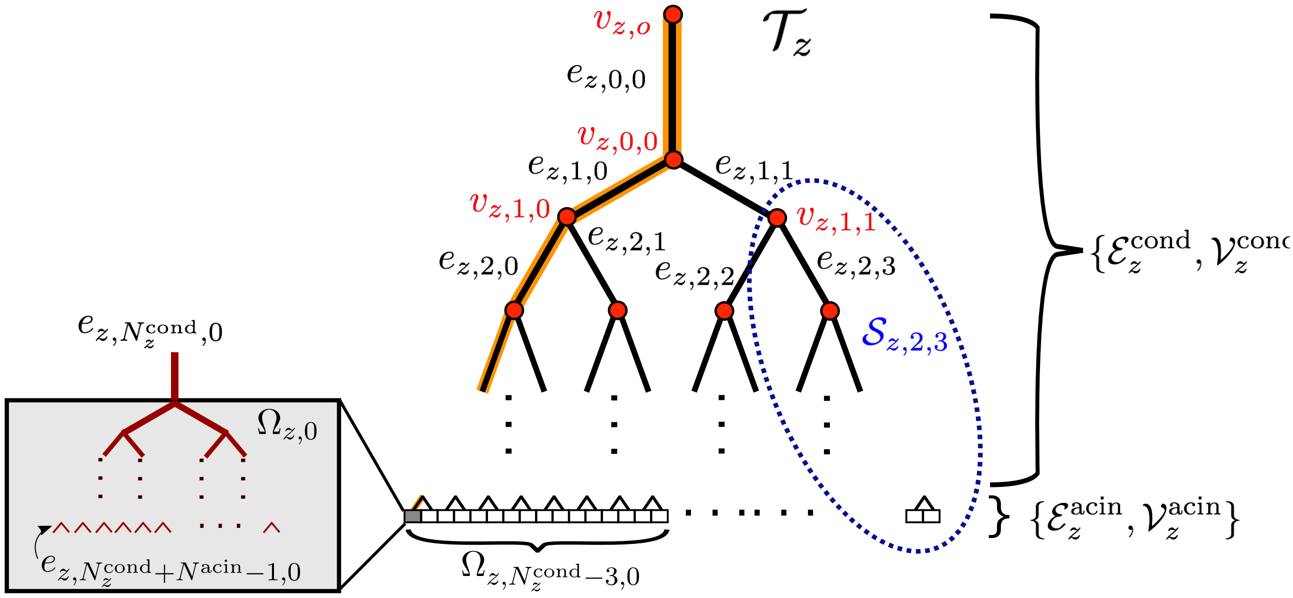

Figure SF1: Sketch of the notation used to identify branches and acini in the SBD tree $\mathcal{T}_z$. Branches $e_{z,j,k}$ (indexed by generation j and branch number k from left to right) form the edges of the network of tubes. The tree terminates in the acini $\Omega_{z,\alpha}$, and groups of these units are identified by their common ancestor branch (as shown for $\Omega_{z,N_z^{\text{cond}}-3,0}$). Paths from the root vertex v_z, o to an acinar unit are labelled $\mathcal{P}_{z,o,\alpha}$ (as highlighted in orange for $\mathcal{P}_{z,o,0}$). Subtrees $\mathcal{S}_{z,j,k}$ consist of all edges and vertices descended from vertex $v_{z,j-1,\lfloor k/2 \rfloor}$ (as highlted by blue dotted ring for $\mathcal{S}_{z,2,3}$). When calculating gas transport, we consider the acinar branches $\Omega_{z,\alpha}$ from generations $N_z^{\text{cond}} \leq j < N_z^{\text{cond}} + N^{\text{acin}}$ (shown for $\Omega_{z,0}$ in inset).

constitute a fraction of the lung parenchyma. The parenchymal volume associated with $\mathcal{T}_z$ is denoted $\Omega_z = \left\{ \Omega_{z,\alpha} \subset \mathbb{R}^3 \mid v_{z,N_z^{\text{cond}}-1,\alpha} \in \Gamma_z \right\}$ such that each terminal vertex is connected to a single acinus $\Omega_{z,\alpha}$. We further denote the subset of Ω_z fed by $e_{z,j,k}$ as $\Omega_{z,j,k}$ such that $\Omega_{z,j,k} = \left\{ \Omega_{z,\alpha} \mid k 2^{N_z^{\text{cond}}-1-j} \leq \alpha < (k+1) 2^{N_z^{\text{cond}}-1-j} \right\}$. This notation is outlined in figure SF1.

1.2 Ventilation model

We have assumed Poiseuille flow in all conducting airways (represented by the edges $e_i \in \mathcal{E}^{\text{cond}}$ where $\mathcal{E}^{\text{cond}} = \{\mathcal{E}_{PA}, \bigcup_{\mathcal{T}_z \in \mathcal{L}} \mathcal{E}_z^{\text{cond}}\}$). The pressure-drop relation is

$$P_{i_m} - P_i = \frac{8\pi\mu l_i}{a_i^2} q_i(t), \tag{1}$$

where μ is the air viscosity, q_i is the rate of flux through branch e_i and P_i is the pressure at vertex v_i where we have used i to refer to either a branch in a SBD tree $(i) = (z, j, k)$ or in the proximal airways. The vertex v_{i_m} is the parent vertex of edge e_i. This relation underestimates the branch resistance as it does not take into account inertial effects which are significant at all but the most distal branches of the tree. However non-linear pressure-drop models (e.g. [2]) are compatible with the approach outlined here.

At breathing frequency air is approximately incompressible, so conservation of mass dictates

$$q_i(t) = \sum_{\Omega_{z,\alpha} \in \Omega_i} \dot{V}_{z,\alpha}(t), \tag{2}$$

where the volume of gas in $\Omega_{z,\alpha}$ is $V_{z,\alpha}$ and the dot is used to indicate derivatives with respect to time t. If a proximal airway, Ω_i denotes the union of all Ω_z downstream of e_u.

Substituting equation (2) into (1) gives a set of equations describing the flux into each acinus. Using this, the pressure-drop relation along the path $\mathcal{P}_{\text{mouth},(z,\alpha)} \in \mathcal{L}$, which connects the mouth vertex v_{mouth} (at the proximal end of the tracheal branch) to acinus $\Omega_{z,\alpha}$, is calculated by summing (1) along each $e_i \in \mathcal{P}_{\text{mouth},(z,\alpha)}$. Repeating this for each $\Omega_{z,\alpha} \in \Omega$ gives a system of $n_{\text{acin}} \equiv \sum_{\mathcal{T}_z \in \mathcal{L}} 2^{N_z^{\text{cond}}}$ coupled Ordinary Differential Equations (ODEs) (*i.e.* one equation for each acinus). In matrix notation these are

$$P_{\text{mouth}}\mathbf{1} - \mathbf{P}(t) = \mathbf{R}_{\text{cond}}\dot{\mathbf{V}}(t), \tag{3}$$

where $\mathbf{P}, \mathbf{V} \in \mathbb{R}^{n_{\text{acin}}}$ have entries $P_{z,\alpha}$ and $V_{z,\alpha}$ corresponding to the pressure and volume for each $\Omega_{z,\alpha} \in \mathcal{L}$. We have assumed that the pressure at vertex $v_{z,N_z^{\text{cond}}-1,\alpha}$ is equal to the gas pressure in the connected acinus $\Omega_{z,\alpha}$. The vector $\mathbf{1}$ is defined as $\mathbf{1} = (1,\dots,1)^T \in \mathbb{R}^{n_{\text{acin}}}$ and the elements of the symmetric resistance matrix $\mathbf{R}_{\text{cond}} \in \mathbb{R}^{n_{\text{acin}}} \times \mathbb{R}^{n_{\text{acin}}}$ [3] are

$$\hat{\mathbf{e}}_{z_1,\alpha_1}^T \mathbf{R}_{\text{cond}} \hat{\mathbf{e}}_{z_2,\alpha_2} = \sum_{e_{j,k} \in \mathcal{P}_{\text{mouth},(z_1,\alpha_1)} \cap \mathcal{P}_{\text{mouth},(z_2,\alpha_2)}} r_i, \tag{4}$$

where the basis vector $\hat{\mathbf{e}}_{z,\alpha} = (0,\dots,0,1,0,\dots,0)^T \in \mathbb{R}^{n_{\text{acin}}}$ is non-zero for the element corresponding to $V_{z,\alpha}$ such that $\hat{\mathbf{e}}_{z,\alpha}^T \mathbf{V} = V_{z,\alpha}$.

To simulate ventilation a constitutive relation is required to determine pressure in the acini Ω. Each acinus $\Omega_{z,\alpha}$ is comprised of a network of alveolar ducts and sacs embedded within the fibrous lung parenchyma. We represent the dynamics of this complex medium with a simple visco-elastic volume, such that each acinus has linear elasticity $K_{z,\alpha}$ and resistance $R_{z,\alpha}$ such that

$$P_{z,\alpha} = \left(P_{\text{mouth}} + P_{z,\alpha}^{(\text{pl})}(t) - P_{z,\alpha}^{(\text{pl0})}\right) + K_{z,\alpha}(V_{z,\alpha}(t) - V_{z,\alpha}^*) + R_{z,\alpha}\dot{V}_{z,\alpha}(t), \tag{5}$$

where $V_{z,\alpha}^*$ is the resting volume of $\Omega_{z,\alpha}$, $P_{z,\alpha}^{(\text{pl})}$ is the intrapleural pressure acting on $\delta\Omega_{z,\alpha}$, and $P_{z,\alpha}^{(\text{pl0})}$ is the reference stationary value of the intrapleural pressure (when $V_{z,\alpha} = V_{z,\alpha}^*$ and $\dot{V}_{z,\alpha} = 0$). In general, the local intrapleural pressure $P_{z,\alpha}^{(\text{pl0})}$ can depend on the volumes and inflation rates of the surrounding acini. We have considered only the simplest case here $P_{z,\alpha}^{(\text{pl})}(t) = P_{\text{pl}}(t)$ and $P_{z,\alpha}^{(\text{pl0})} = P_{\text{pl0}}$ for all $\Omega_{z,\alpha} \in \mathcal{L}$, such that the pressure applied to all acini have identical properties.

Substituting equation (5) into equation (3) gives the following set of linear equations for the full ventilation dynamics,

$$\left(\mathbf{R}_{\text{cond}} + \mathbf{R}_{\text{acin}}\right)\dot{\mathbf{V}}(t) + \mathbf{K}\left(\mathbf{V}(t) - \mathbf{V}^*\right) = \left(P_{\text{pl0}} - P_{\text{pl}}(t)\right)\mathbf{1}, \tag{6}$$

where $\mathbf{V}^* \in \mathbb{R}^{n_{\text{acin}}}$ is the vector of resting volumes $V_{z,\alpha}^*$ of the parenchymal sub-units and the matrix $\mathbf{K} \in \mathbb{R}^{n_{\text{acin}}} \times \mathbb{R}^{n_{\text{acin}}}$ is diagonal with entries $K_{z,\alpha}$, and similarly $\mathbf{R}_{\text{acin}} \in \mathbb{R}^{n_{\text{acin}}} \times \mathbb{R}^{n_{\text{acin}}}$ is diagonal with entries $R_{z,\alpha}$. In a more complex description with linear mechanical coupling between acini, the matrices $\mathbf{R}_{\text{acin}}$ and $\mathbf{K}$ could have off-diagonal entries. The linear ventilation equations used here have a direct electrical analogue where the acinus is modelled a capacitor and resistor in series, while airways are a network of resistors with current q and potential P at the vertices.

1.3 Transport model

Gas transport on the network is modelled as one-dimensional advection-diffusion in each airway, with transport into the alveolar sacs accounted for by-a two cylinder model adopted in [4, 5]. The volume fraction of inert tracer gas on edge $e_i \in \mathcal{L}$ (using i generically to refer to any index or indices that refer to a branch in the network) is modelled as a one-dimensional (cross-sectionally averaged) concentration field $c_i(x,t)$ where x is the distance along the edge from its parent vertex $v_{i'}$. Thus, in this convention, inspiratory (expiratory) flows are those directed down (up) the tree and are positive (negative).

The ventilation model in the previous section treats the parenchyma as a three-dimensional visco-elastic domain connected to the end of the tree. However, the detailed internal structure of the acinus is important to modelling gas transport. Therefore, we use the idealised gas-transport equations outlined in [6] which represents each acinus $\Omega_{z,\alpha}$ by the sub-tree $\mathcal{S}_{z,N^{\text{cond}},\alpha} \equiv \mathcal{S}_{z,\alpha}^{\text{acin}}$ such that $\bigcup_{\alpha\in\mathcal{T}_z} \mathcal{S}_{z,\alpha}^{\text{acin}} = \{\mathcal{E}_z^{\text{acin}}, \mathcal{V}_z^{\text{acin}}\}$.

In each acinus $\Omega_{z,\alpha}$ the tubes are lined with alveoli, which is accounted for by writing the total tube cross-section as $A_i(t) = a_i + n_i^{(\text{sac})} V^{(\text{sac})}(t)/l_i \; \forall e_i \in \mathcal{S}_{z,\alpha}^{\text{acin}}$ where a_i is the cross-section of the alveolar duct (assumed rigid) and the second term is the alveolar sac volume per unit length on the branch e_i. In particular n_i^{sac} is the number of alveolar sacs lining e_i and $V^{\text{sac}}(t)$ is the volume of each alveolar sac in the acinus. Following [6], we have assumed that all alveolar sacs in a given acinus are identical and inflate at the same rate. Average anatomical data [7] suggest that the number of alveoli lining a branch in the acinus is proportional to the duct surface area $l_i\sqrt{a_i}$ with a correction factor $0 < \Phi_i \le 1$ which depends only on the branch generation relative to the terminal bronchiole generation $j - N_z^{\text{cond}}$ (a detailed parameter list is given in S1 Table). Therefore the total branch cross-section in each acinus can be written

$$A_i(t) = a_i + V_{z,\alpha}(t)\Phi_i\sqrt{a_i} \bigg/ \left(\sum_{e_{\tilde{i}} \in \mathcal{S}_{z,\alpha}^{\text{acin}}} \Phi_{\tilde{i}} l_{\tilde{i}} \sqrt{a_{\tilde{i}}} \right) \quad \forall e_i \in \mathcal{S}_{z,\alpha}^{\text{acin}}, \forall \mathcal{S}_{z,\alpha}^{\text{acin}} \in \mathcal{L}. \tag{7}$$

Note that we consistently define $A_i = a_i$ for all branches in the conducting network $e_i \in \mathcal{E}^{\text{cond}}$ where there are no alveolar sacs.

We approximate the gas concentration as well-mixed in the radial direction, but unable flow directly between sacs in the acinus. Within a conducting branch the flux is taken to be

$$F_i(x,t) = a_i \left[u_i(t)c_i(x,t) - D_i(t)\frac{\partial c_i(x,t)}{\partial x} \right] \quad \forall e_i \in \mathcal{E}^{\text{cond}}, \tag{8}$$

where $u_i = q_i/a_i$ is the flow velocity in the tube and the diffusion coefficient $D_i(x,t) = D_0 + 2Cu_i(x,t)\sqrt{a_i/\pi}$ is corrected for Taylor-like dispersion in a branching tube network, where $C = 1.08$ for inspiration and $C = 0.37$ for expiration [8]. In the acinar branches, we assume no flow-dependent dispersion rate as we know of no such relations derived for this complex expanding geometry and the flux relation is

$$F_i(x,t) = a_i u_i(x,t)c_i(x,t) - D_0\left[\phi A_i(t) + (1-\phi)a_i\right]\frac{\partial c_i(x,t)}{\partial x} \quad \forall e_i \in \mathcal{E}_z^{\text{acin}}, \forall \mathcal{E}_z^{\text{acin}} \in \mathcal{L}, \tag{9}$$

where ϕ is a phenomenological parameter that sets the fraction of alveolar sac cross-section that is involved in diffusion [5] (see S1 Table).

Mass conservation and incompressibility conditions for branch $e_{j,k}$ are given by

$$\frac{\partial}{\partial t}\left[A_i(t)c_i(x,t)\right] = -\frac{\partial F_i(x,t)}{\partial x} - G_i(x,t,c_{j,k}) \quad \forall e_i \in \mathcal{L}, \tag{10}$$

$$\frac{1}{a_i}\frac{\partial A_i(t)}{\partial t} + \frac{\partial u_i(x,t)}{\partial x} = 0 \quad \forall e_i \in \mathcal{L}. \tag{11}$$

We have included here a generic uptake/source term G_i to account for gas exchange in the alveoli, but for the purposes of inert gas washout tests we assume that uptake is negligible and $G_i = 0$ throughout. Equation (11) defines the air velocity everywhere, given that it is zero at all endpoints $v_{z,N_z^{\rm cond}+N_z^{\rm acin}-1,k} \in \mathcal{T}_z$ for all $\mathcal{T}_z \in \mathcal{L}$ and A_i is calculated consistently by equation (7). This, in turn, is calculated from the acinar volumes computed in equation (6). Flux conservation implies continuity of the concentration field at branching points, but a discontinuity in the gradient.

1.4 Model M: Mean-path reduction

If all the airways in each generation of a SBD sub-tree are assumed to be identical, then these sub-trees can be replaced by a single representative path. We call this a mean-path reduction when the airway and acinus properties of this representative path are is an average of the real structure (as used in Weibel's model A [9] for example).

Replacing a particular sub-tree $\mathcal{S}_{z,j',k'}$ with a mean-path changes how we represent this in the network $\mathcal{T}_z$ (figure SF2). As all branches in the sub-tree are assumed to behave in an identical manner, they can be simply represented by a single edge or series of edges. The network becomes $\bar{\mathcal{T}}_z = (\mathcal{T}_z \setminus \mathcal{S}_{z,j',k'}) \cup \bar{\mathcal{S}}_{z,j',k'}$ (see figure SF2) where

$$\bar{\mathcal{S}}_{z,j',k'} = v_{z,j'-1,\lfloor k'/2\rfloor} \cup \left\{\left\{\bar{e}_{z,j}^{(j',k')}, \bar{v}_{z,j}^{(j',k')}\right\} \mid j' \leq j < N_z^{\rm cond} + N^{\rm acin}\right\}. \tag{12}$$

Each mean-path edge $\bar{e}_{z,j}^{(j',k')}$ and vertex $\bar{v}_{z,j}^{(j',k')}$ (signified by the over-bar) represents all the branches in generation j of the original sub-tree $\mathcal{S}_{z,j',k'}$. The total duct cross-section associated with $\bar{e}_{z,j}^{(j',k')}$ is $\bar{s}_{z,j}^{(j',k')} = 2^{j-j'}\bar{a}_{z,j}^{(j',k')}$ where $\bar{a}_{z,j}^{(j',k')}$ is the mean branch cross-section of generation j in $\mathcal{S}_{z,j',k'}$. The effective resistance of edge $\bar{e}_{z,j}^{(j',k')}$ is that of $2^{j-j'}$ Poiseuille resistors in parallel such that

$$\bar{r}_{z,j}^{(j',k')} = \frac{8\pi\mu\bar{l}_{z,j}^{(j',k')}}{2^{j-j'}(\bar{a}_{z,j}^{(j',k')})^2}. \tag{13}$$

this effective resistance is computed from the mean geometry, which is different to the mean resistance of branches with different geometries. The pressure-flow relation over the edges $\bar{e}_{z,j}^{(j',k')} \in \bar{\mathcal{E}}^{\rm cond}$ is

$$\left(\bar{R}_{z,j',k'}^{\rm acin} + \sum_{j=j'}^{N_z^{\rm cond}} \bar{r}_{z,j}^{(j',k')}\right)\dot{V}_{z,j',k'} + K_{z,j',k'}\left(V_{z,j',k'} - V_{z,j',k'}^{\star}\right) = P_{z,j'-1,\lfloor k'/2\rfloor} - \bar{P}_{N_z^{\rm cond}}^{(j',k')}, \tag{14}$$

where the bulk acinus properties are

$$K_{z,j',k'} = 4^{j'-N_z^{\rm cond}} \sum_{\alpha\in\Omega_{z,j',k'}} K_{z,\alpha}\,, \quad R_{z,j',k'}^{\rm acin} = 4^{j'-N_z^{\rm cond}} \sum_{\alpha\in\Omega_{z,j',k'}} R_{z,\alpha},$$
$$V_{z,j',k'}^{\star} = \sum_{\alpha\in\Omega_{z,j',k'}} V_{z,\alpha}^{\star}. \tag{15}$$

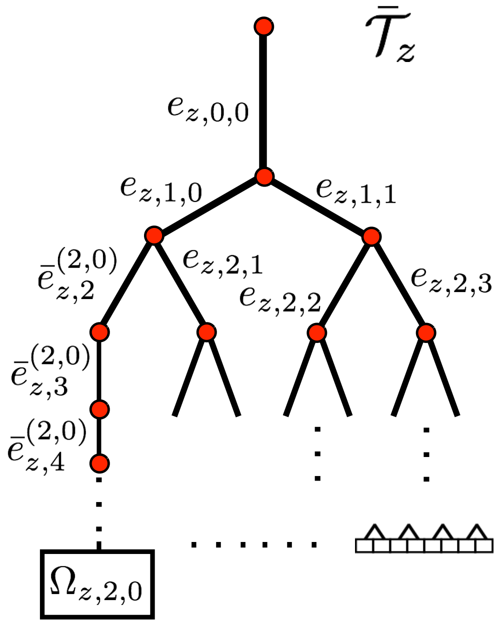

Figure SF2: A mean-path tree network $\bar{\mathcal{T}}_z$ with sub-tree $\mathcal{S}_{z,2,0}$ replaced by a mean-path.

Thus, the mean-path represents the whole sub-tree $\mathcal{S}_{z,j',k'}$ with a single path terminating in a collection of identical acini with collective elastance $K_{z,j',k'}$ and total alveolar volume $V_{z,j',k'}$. The resistances remain unchanged for all $e_{z,j,k} \in \bar{\mathcal{T}} \setminus \bar{\mathcal{S}}_{z,j',k'}$, *i.e.* those edges not included in the mean-path.

The mean-path pressure-drop equation then replaces the subtree $\mathcal{S}_{z,j',k'}$ in the set of ventilation ODEs, which is solved as before (see section 1.2). Conservation of mass and incompressibility on sub-tree $\bar{\mathcal{S}}_{z,j',k'}$ give the following mean-path transport equation

$$\frac{\partial}{\partial t}\left[S_{z,j}(t)\bar{c}_{z,j}(\bar{x},t)\right] + \frac{\partial}{\partial \bar{x}}\left[s_{z,j}\bar{u}_{z,j}(\bar{x},t)\bar{c}_{z,j}(\bar{x},t)\right] = \frac{\partial}{\partial \bar{x}}\left[\bar{D}_{z,j}s_{z,j}(\bar{x},t)\frac{\partial \bar{c}_{z,j}(\bar{x},t)}{\partial \bar{x}}\right], \tag{16}$$

$$\frac{1}{s_{z,j}}\frac{\partial S_{z,j}(t)}{\partial t} + \frac{\partial \bar{u}_{z,j}(x,t)}{\partial x} = 0. \tag{17}$$

where we have omitted the superscripts (j',k') on all airway properties for clarity. The total cross-section of all the mean-path ducts plus alveoli is $S_{z,j} = 2^{j-j'}\bar{A}_{z,j}$. The distance coordinate $\bar{x} = \sum_{\tilde{j}=j'}^{j-1}\bar{l}_{z,\tilde{j}} + \bar{l}_{z,j}\left(x/l_{z,j,k}\right)$, for any $\left\{l_{z,j,k} \mid e_{z,j,k} \in \mathcal{S}_{z,j',k'}\right\}$ is scaled by the mean branch length. The mean-path velocity $\bar{u}_{z,j}(x,t)$ and the effective diffusion coefficient $\bar{D}_{z,j}(x,t)$ are computed from the mean airway geometry. There is again a discontinuity in cross-section at the generation boundaries, which in turn implies a discontinuity in concentration gradient so that diffusive flux is conserved, and a discontinuity in flow rate so that concentration is assumed consistent. This is dealt with numerically using the finite volume method outlined in section 2.

In model M, we represent each of the SBD trees $\mathcal{T}_z \in \mathcal{L}$ with a mean-path representation ($\mathcal{T}_z \to \bar{\mathcal{S}}_{z,0,0}$), equivalent to a 'trumpet' model. The baseline (unconstricted) properties of the airways in the different lung regions of model M are set to be equal for each Strahler order. We have chosen $N^{\rm cond}_{RU} = N^{\rm cond}_{LU} = N^{\rm cond}_{RL_{\rm maj}} = N^{\rm cond}_{LL_{\rm maj}} = 13$, $N^{\rm cond}_{RM} = 12$ and $N^{\rm cond}_{RL_{\rm min}} = N^{\rm cond}_{LL_{\rm min}} = 11$. Conducting airway diameter and length have a fixed ratio $LD_{\rm cond}$ and each airway scales with its parent branch according

to the Murray–Hess law

$$\bar{a}_{z,j} = \pi \left(\frac{\bar{l}_{z,j}}{2LD_{\text{cond}}} \right)^2, \tag{18}$$

$$\bar{l}_{z,j+1} = \lambda \bar{l}_{z,j} \quad \forall \bar{e}_{z,j} \in \bar{\mathcal{E}}_z^{\text{cond}} \quad \forall \mathcal{T}_z \in \mathcal{L} \tag{19}$$

where $\lambda \approx 2^{-1/3}$. The volume of the conducting airways is fitted to their total volume $V_D - V_{\text{PA}}$ where V_D is the anatomical dead-space (excluding the mouth) and V_{PA} is the volume of the airways in $\mathcal{E}^{\text{PA}}$, such that

$$\bar{l}_{RU,0} = \left(\frac{4LD_{\text{cond}}^2 \left(V_D - V_{\text{PA}} \right)}{\pi \sum_{\mathcal{T}_z \in \mathcal{L}} \sum_{j=0}^{N_z^{\text{cond}}-1} \lambda^{3(12-j)}} \right)^{1/3}, \tag{20}$$

and $\bar{l}_{z,0} = \lambda^{13-N_z^{\text{cond}}} \bar{l}_{RU,0}$. All acini have been treated as identical in the baseline model with constant length and area scaling between parent and daughter ducts of λ_{acin} and a fixed ratio of length to diameter LD_{acin},

$$\bar{a}_{z,j} = \pi \left(\frac{\bar{l}_{z,j}}{2LD_{\text{acin}}} \right)^2, \tag{21}$$

$$\bar{l}_{z,j+1} = \lambda_{\text{acin}} \bar{l}_{z,j} \quad \forall \bar{e}_{z,j} \in \bar{\mathcal{E}}_z^{\text{acin}} \quad \forall \mathcal{T}_z \in \mathcal{L}. \tag{22}$$

The acinar ducts constitute a fraction $V_{\text{duct}}/V_{\text{acin}}$ of the total acinar volume and so fitting the volume to the physiological parameters gives

$$\bar{l}_{z,N_z^{\text{cond}}} = \left(\frac{4LD_{\text{acin}}^2 V_{\text{duct}} \left(V_{\text{FRC}} - V_{\text{D}} \right)}{\pi V_{\text{acin}} \sum_{\mathcal{T}_z \in \mathcal{L}} \sum_{j=N_z^{\text{cond}}}^{N_z^{\text{acin}}} \lambda^{3j}} \right)^{1/3} \quad \forall \mathcal{T}_z \in \mathcal{L}. \tag{23}$$

This representation in results in $2^{15} + 2^{13}$ terminal bronchioles, which is an overestimate of Weibel's estimate (∼30,000) [10], but accounts well for the relative lobe volumes [1].

Constrictions applied to airways only change their cross-section. In generating the data for Figs 4(a,c,e), 5, 6, and 7 in the main text, constrictions are applied to all airways in a given generation range of $\mathcal{T}_{\text{RM}}$, and so $\mathcal{T}_{\text{RM}}$ is still represented by the mean-path $\bar{\mathcal{S}}_{\text{RM},0,0}$ (as shown in S1 video) with the relevant mean-path cross-sections $\bar{a}_{\text{RM},j}$ altered accordingly. For Figs 4(b,d,f) in the main text, constrictions are spread across the network, and in this case mean-paths are only used for sub-trees consisting of generations of airways that are either all constricted or all unconstricted (see S2 video for the resulting network structure).

1.5 Model P: Limit of weak heterogeneity

In model P, the geometry of the airways and acinar elastance are described in terms of deviations from the mean-path representation of model M (where $\mathcal{T}_z \to \bar{\mathcal{S}}_{z,0,0}$). The superscript $(0,0)$ is implied for all mean-path edge properties, and hence is omitted throughout this section for clarity ($e.g.$ $\bar{a}_{z,j}^{(0,0)} \equiv \bar{a}_{z,j}$). We write $a_{z,j,k} = \bar{a}_{z,j}(1 + \epsilon_{z,j,k}^{(a)})$, $l_{z,j,k} = \bar{l}_{z,j}(1 + \epsilon_{z,j,k}^{(l)})$ and $K_{z,\alpha} = 2^{-N_z^{\text{cond}}} K_{z,0,0}(1 + \epsilon_{z,j,k}^{(K)})$ such that the ϵ parameters measure the individual airway deviation from the mean-path value. In the limit of

small perturbations the resistance can be approximated as linear with respect to the ϵ parameters

$$r_{z,j,k} \approx \bar{r}_{z,j} \left(1 + \epsilon^{(l)}_{z,j,k} - 2\epsilon^{(a)}_{z,j,k}\right). \tag{24}$$

In this limit of $\epsilon \ll 1$, the change in any output variable $g \in \{A, c, u, D\}$ in the tree $\mathcal{T}_z$ can be expressed as a linear superposition of the contributions from each perturbation

$$\Delta g_{z,j,k} \equiv g_{z,j,k} - \bar{g}_{z,j} = \sum_p \sum_{\mathcal{T}_{z'} \in \mathcal{L}} \sum_{e_{j',k'} \in \mathcal{T}_{z'}} \delta g^{(p_{z',j',k'})}_{z,j,k} \epsilon^{(p)}_{z',j',k'} + O(\epsilon^2). \tag{25}$$

The linear sensitivity functions $\delta g^{(p_{z',j',k'})}_{z,j,k}$ are degenerate with respect to branch numbers k and k' due to symmetry at zeroth order. First, if the measured variable is in a different mean-path to the perturbation ($z' \neq z$, including the proximal airways) then the response is independent of branch number (as all branches in a given generation of a mean-path are identical at zeroth order). Secondly, if the measured variable is in the same mean-path as the perturbation ($z' = z$), then the linear sensitivity is dependent only on the generation of the lowest common ancestor (LCA) of $e_{z,j,k}$ and $e_{z,j',k'}$ and their respective generations. These can be summarised by

$$\delta g^{(p_{z',j',k'})}_{z,j,k} = \delta_{z,z'} \delta g^{(p,j',j_{\rm LCA})}_{z,j} + (1 - \delta_{z,z'}) \delta g^{(p,z',j')}_{z,j}. \tag{26}$$

The parameter $j_{\rm LCA} \equiv j_{\rm LCA}(z, j, k, j', k')$ is the generation number of the LCA of branches $e_{z,j,k}$ and $e_{z,j',k'}$,

$$j_{\rm LCA}(z, j, k, j', k') = \max \left\{ \tilde{j} \mid e_{z,\tilde{j},\tilde{k}} \in \mathcal{P}_{z,o,k2^{N_z^{\rm cond} - j}} \cap \mathcal{P}_{z,o,k'2^{N_z^{\rm cond} - j'}} \right\}. \tag{27}$$

Thus, linear sensitivities need only be calculated for one perturbation to each generation of the SBD networks $\mathcal{T}_{z'}$ (see Fig 2 in the main text). Without loss of generality, we choose to always perturb the left-most branch in each case, *i.e.* $k' = 0$. The response to the perturbation of $e_{z',j',0}$ on $\mathcal{L}$ is calculated by replacing the sub-trees $\mathcal{S}_{z',j,k} \in \mathcal{S}_{z',j',0} \cup \left\{ \mathcal{S}_{\tilde{j},1} \mid 0 < \tilde{j} \leq j' \right\}$ with mean-paths, as shown in figure SF3. We use $\bar{\mathcal{T}}_{z',j'}$ to denote this particular tree, and $\mathcal{L}_{z',j'}$ to denote the resulting whole lung network, that is used to simulate the response to a single perturbation at $e_{z',j',0}$.

The linear perturbation to the ventilation equation (6) on $\mathcal{L}_{z',j'}$ is

$$\begin{aligned} &\left(\bar{\mathbf{R}}^{(\rm cond)}_{z',j'} + \bar{\mathbf{R}}^{(\rm acin)}_{z',j'}\right) \Delta\dot{\mathbf{V}} + \bar{\mathbf{K}}_{z',j'} \Delta\mathbf{V} \\ &+ \left(\Delta r_{z',j',0} \dot{V}_{z',j',0} + \Delta K_0 V_{z',j',0} \delta_{j',N_z^{\rm cond}}\right) \hat{\mathbf{e}}_{z',j',0} = -\Delta P^{(\rm pl)}(t) \mathbf{1}, \end{aligned} \tag{28}$$

where the resistance and stiffness matrices $\bar{\mathbf{R}}_{z',j'}, \bar{\mathbf{K}}_{z',j'} \in \mathbb{R}^{7+j'} \times \mathbb{R}^{7+j'}$ consist of the mean-path parameters of order $O(\epsilon^0)$ on the network $\mathcal{L}_{z',j'}$, and $\Delta\mathbf{V} = (\Delta V_{z1\neq z',0,0}, \ldots, \Delta V_{z6\neq z',0,0}, \Delta V_{z',1,1}, \ldots, \Delta V_{z',j',0}, \Delta V_{z,'j',1})$ is the vector of $O(\epsilon^1)$ changes to the acinar volumes in $\mathcal{L}_{z',j'}$. The first six entries are the volume changes in the unperturbed SBD trees ($\mathcal{T}_z$ for $z \neq z'$), and the remainder are sub-trees of the perturbed tree $\bar{\mathcal{T}}'_{z',j'}$ (demonstrated in figure SF3). The perturbed airway (or acinus) contributes to a single entry only in both of these tensors, namely the diagonal entry associated with perturbed sub-tree $\bar{\mathcal{S}}_{z',j',0}$ shown by the basis vector $\mathbf{e}_{z',j',0}$ such that $\hat{\mathbf{e}}^T_{z',j',k'} \Delta\mathbf{V} = \Delta V^{z',j',0}$. Equation (28) is solved for $\Delta\mathbf{V}$ at each time step by inverting the equation under the constraint that the flow rate at the mouth is prescribed $\mathbf{1}^T \Delta\dot{\mathbf{V}} = 0$, which sets the pressure perturbation $\Delta P^{(\rm pl)}(t)$.

The solution of equation (28) gives the perturbed volumes of the parenchymal units, which are

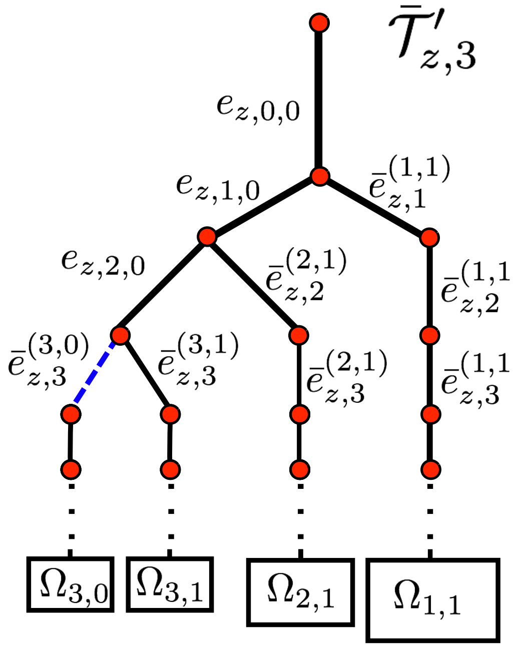

Figure SF3: Perturbed network $\bar{\mathcal{T}}'_{z'}$ for a perturbation of branch $e_{3,0}$ (dashed blue line) resulting in four terminal sub-trees which account for $1/8, 1/8, 1/4, 1/2$ of the total acinar volume of $\mathcal{T}_z$ respectively from left to right in the diagram.

then distributed on to the tree using equation (7) to linear order in the perturbations

$$\Delta A_i = \Delta a_i + \Bigg\{ \Delta V_{z,\alpha}(t)\Phi_i\sqrt{\bar{a}_i} + V_{z,\alpha}(t)\Phi_i\frac{\Delta a_i}{2\sqrt{\bar{a}_i}} - \left[\sum_{e_{\tilde{i}}\in\mathcal{S}^{\text{acin}}_{z,\alpha}} \Phi_{\tilde{i}}\left(\Delta l_{\tilde{i}}\sqrt{\bar{a}_{\tilde{i}}} + \bar{l}_{\tilde{i}}\frac{\Delta a_{\tilde{i}}}{2\sqrt{\bar{a}_{\tilde{i}}}}\right)\right] \Bigg/ \left[\sum_{e_{\tilde{i}}\in\mathcal{S}^{\text{acin}}_{z,\alpha}} \Phi_{\tilde{i}}\bar{l}_{\tilde{i}}\sqrt{\bar{a}_{\tilde{i}}}\right]\Bigg\} \Bigg/ \left(\sum_{e_{\tilde{i}}\in\mathcal{S}^{\text{acin}}_{z,\alpha}} \Phi_{\tilde{i}}\bar{l}_{\tilde{i}}\sqrt{\bar{a}_{\tilde{i}}}\right) \quad (29)$$

$$\forall e_i \in \mathcal{S}^{\text{acin}}_{z,\alpha}\ ,\ \forall \mathcal{S}^{\text{acin}}_{z,\alpha} \in \mathcal{L}_{z',j'}.$$

Finally, the $O(\epsilon^1)$ concentration field is computed by the linearised form of (10) as

$$\frac{\partial}{\partial t}\left[\bar{c}_i\bar{S}_i\left(\frac{\Delta c_i}{\bar{c}} + \frac{\Delta A_i}{\bar{A}_i}\right)\right] + \frac{\partial}{\partial \bar{x}}\left[\bar{u}_i\bar{s}_i\bar{c}_i\left(\frac{\Delta u_i}{\bar{u}_i} + \frac{\Delta a_i}{\bar{a}_i} + \frac{\Delta c_i}{\bar{c}_i}\right)\right] = \frac{\partial}{\partial \bar{x}}\left[\bar{D}_i\bar{s}_i\frac{\partial \bar{c}_i}{\partial \bar{x}}\left(\frac{\Delta D_i}{\bar{D}_i} + \frac{\Delta s_i}{\bar{s}_i} + \frac{\partial \Delta c_i/\partial \bar{x}}{\partial \bar{c}_i/\partial \bar{x}} - \frac{\Delta l_i}{\bar{l}_i}\frac{\partial \bar{c}_i}{\partial \bar{x}}\right)\right] + \frac{\Delta l_i}{\bar{l}_i}\frac{\partial}{\partial \bar{x}}\left(\bar{u}_i\bar{s}_i\bar{c}_i\right) - \frac{\Delta l_i}{\bar{l}_i}\frac{\partial}{\partial \bar{x}}\left(\bar{D}_i\bar{s}_i\frac{\partial \bar{c}_i}{\partial \bar{x}}\right), \quad (30)$$

$$\frac{\partial \Delta S_i}{\partial t} + \frac{\partial}{\partial x}\left[\bar{s}_i\bar{u}_i\left(\frac{\Delta u_i}{\bar{u}_i} + \frac{\Delta s_i}{\bar{s}_i}\right)\right] = 0. \forall e_i \in \mathcal{L}_{z',j'} \quad (31)$$

The quantities Δs_i and ΔS_i are the absolute changes in total cross-sectional area in airway e_i. The branch length change Δl_i is only non-zero if that branch contains a length perturbation ($\epsilon^{(l)}_i \neq 0$).

1.6 Calculating sensitivity to global parameters

The linear sensitivities of model variables to airway and acinar properties can be combined to measure sensitivity to global model parameters. Here we use them to probe the sensitivity of LCI to changes in the length-diameter ratio of the conducting airways ($LD_{\rm cond}$, assuming fixed airway volume), the conducting airway volume (V_D, assuming fixed length-diameter ratio) and the total lung elastance. To perturb these three properties, we use perturbation values respectively

$$\epsilon^{(l)}_{z,j,k} = \frac{2}{3}\frac{\Delta LD_{\rm cond}}{LD_{\rm cond}}\,,\ \epsilon^{(a)}_{z,j,k} = -\epsilon^{(l)}_{z,j,k} \quad \forall e_{z,j,k} \in \mathcal{E}^{\rm cond}_z,\ \forall \mathcal{T}_z \in \mathcal{L}, \tag{32}$$

$$\epsilon^{(l)}_{z,j,k} = \frac{1}{3}\frac{\Delta V_D}{V_D}\,,\ \epsilon^{(a)}_{z,j,k} = 2\epsilon^{(l)}_{z,j,k} \quad \forall e_{z,j,k} \in \mathcal{E}^{\rm cond}_z,\ \forall \mathcal{T}_z \in \mathcal{L}, \tag{33}$$

$$\epsilon^{(K)}_{z,\alpha} = \frac{\Delta K_{\rm lung}}{K_{\rm lung}} \quad \forall \Omega_{z,\alpha} \in \Omega_z,\ \forall \mathcal{T}_z \in \mathcal{L}. \tag{34}$$

This means we can calculate the sensitivity of LCI to these global parameters by substituting equations (32)-(34) into equation (25) with g =LCI to find

$$\delta {\rm LCI}^{(LD_{\rm cond})} = \frac{2}{3}\sum_{\mathcal{T}_z\in\mathcal{L}}\ \sum_{e_{z,j,k}\in\mathcal{E}^{\rm cond}_z}\left(\delta{\rm LCI}^{(l_{z,j,k})} - \delta{\rm LCI}^{(a_{z,j,k})}\right), \tag{35}$$

$$\delta {\rm LCI}^{(V_D)} = \frac{1}{3}\sum_{\mathcal{T}_z\in\mathcal{L}}\ \sum_{e_{z,j,k}\in\mathcal{E}^{\rm cond}_z}\left(\delta{\rm LCI}^{(l_{z,j,k})} + 2\delta{\rm LCI}^{(a_{z,j,k})}\right), \tag{36}$$

$$\delta {\rm LCI}^{(K_{lung})} = \sum_{\mathcal{T}_z\in\mathcal{L}}\ \sum_{\Omega_{z,\alpha}\in\Omega_z}\delta{\rm LCI}^{(K_{z,\alpha})}. \tag{37}$$

The above relations can be reduced using the redundancy relations to

$$\delta {\rm LCI}^{(LD_{\rm cond})} = \frac{2}{3}\sum_{\mathcal{T}_z\in\mathcal{L}}\ \sum_{e_{z,j,k}\in\mathcal{E}^{\rm cond}_z}\left(\delta{\rm LCI}^{(l_{z,j,k})} - \delta{\rm LCI}^{(a_{z,j,k})}\right), \tag{38}$$

$$\delta {\rm LCI}^{(V_D)} = \frac{1}{3}\sum_{\mathcal{T}_z\in\mathcal{L}}\ \sum_{e_{z,j,k}\in\mathcal{E}^{\rm cond}_z}\left(\delta{\rm LCI}^{(l_{z,j,k})} + 2\delta{\rm LCI}^{(a_{z,j,k})}\right), \tag{39}$$

$$\delta {\rm LCI}^{(K_{lung})} = \sum_{\mathcal{T}_z\in\mathcal{L}}\ \sum_{\Omega_{z,\alpha}\in\Omega_z}\delta{\rm LCI}^{(K_{z,\alpha})}. \tag{40}$$

2 Numerical simulation of coupled ventilation and transport

We have numerically integrated the ventilation equation (6) using the Crank–Nicholson scheme

$$\left(\mathsf{R} + {\rm d}t\frac{\mathsf{K}}{2}\right)\mathbf{V}^{n+1} = \left(\mathsf{R} - {\rm d}t\frac{\mathsf{K}}{2}\right)\mathbf{V}^{n} + {\rm d}t\left[\mathsf{K}\mathbf{V}^* + \left(P_{\rm pl0} - \frac{1}{2}\left(P^n_{\rm pl} + P^{n+1}_{\rm pl}\right)\right)\mathbf{1}\right], \tag{41}$$

where dt is the discretised time-step (dt = 0.025s used for all simulations) and the superscripts of the dynamic quantities indicate time-step, *e.g.* $\mathbf{V}^n = \mathbf{V}(t = n{\rm d}t)$. Given a prescribed function for $P_{\rm pl}$, this equation can be solved by direct inversion. We choose to prescribe the net flow instead, and infer the pleural pressure at each time-step due to this constraint. The linear system of equations then

becomes

$$\begin{pmatrix} \mathbf{A} & \mathbf{1} \\ \mathbf{1}^T & 0 \end{pmatrix} \begin{pmatrix} \mathbf{V}^{n+1} \\ \mathrm{d}tP_{\mathrm{pl}}^{n+1}/2 \end{pmatrix} = \begin{pmatrix} \mathbf{B} & -\mathbf{1} \\ \mathbf{1}^T & 0 \end{pmatrix} \begin{pmatrix} \mathbf{V}^{n} \\ \mathrm{d}tP_{\mathrm{pl}}^{n}/2 \end{pmatrix} + \mathrm{d}t \begin{pmatrix} \mathbf{K}\mathbf{V}^* + P_{\mathrm{pl}0}\mathbf{1} \\ \dot{q}_{\mathrm{mouth}}\left((n+1/2)\mathrm{d}t\right) \end{pmatrix} \tag{42}$$

where $\mathbf{A} = \mathbf{R} + \mathrm{d}t\mathbf{K}/2$ and $\mathbf{B} = \mathbf{R} - \mathrm{d}t\mathbf{K}/2$. The bottom line of (42) ensures that the total flow rate at time $t = (n+1/2)\mathrm{d}t$ is set by the prescribed function $\dot{q}_{\mathrm{mouth}}(t)$. This system is solved in C++ using the Eigen [11] factorisation routine 'PartialPivLU'. We initialise the simulation with $\mathbf{V}^0 = \mathbf{V}^*$ and run the ventilation solver for 25 breaths before initiating the simulation of washout to reach a stationary breathing pattern.

At each time step the cross-section of the acinar airways are calculated through equation (7). Note that in general the tree structure may contain mean-path branches, which in turn my contain a whole acinus, or part of single acinus (depending on the tree structure being simulated). To simulate gas transport we discretise the network of edges $e_i \in \mathcal{L}$ into finite volume elements $w_{i,x}$ for $x \in \{0, 1, \ldots, p_i - 1\}$ (see figure SF4) where p_i is the number of volumes that constitute the branch e_i. We define each volume in a branch to have equal length

$$h_{i,x} = l_i/p_i \quad \forall w_{i,x} \in e_i, \tag{43}$$

and choose p_i as the maximum of either $p_{\min}$, the quantity $\lfloor Pe(l_i)/Pe_{\min} \rfloor$, or $\lfloor l_i/(0.025L) \rfloor$ where $Pe(l_i)$ estimates the maximum longitudinal Peclet number of the branch i and $Pe_{\min}$ is the minimum volume element Peclet number. The distance L is the length of any path from the mouth the end point of $\mathcal{T}^{(\mathrm{RL_{maj}})}$. We have chosen $p_{min} = 1$ and $Pe_{\min} = 10$ for conducting branches and $p_{\min} = 4$ and $Pe_{\min} = \infty$ for acinar ducts. Thus each branch in the conducting zone consists of at least one finite volume, and each acinar duct at least four. These values were chosen following tests varying these parameters independently and recording the convergence to a solution (see documentation accompanying code at [12]). We write the cross-sections of branch e_i in terms of s_i and S_i to account for the possibility that e_i is in a mean-path. Each volume element $w_{i,x} \in e_i$ also has equal cross-section, and therefore equal volume. Each volume element $w_{i.x} \in e_i$ has concentration $c_{i,x}$, and velocity $u^{(l)}_{i,x}, u^{(r)}_{i,x}$ and diffusion coefficients $D^{(l)}_{i,x}, D^{(r)}_{i,x}$ defined at the left and right edges. The velocities at time $(n+1/2)\mathrm{d}t$ are calculated through the incompressibility equation as

$$u^{(l),n+1/2}_{i,x} = u^{(r),n+1/2}_{i,x} + \frac{h_{i,x}}{\mathrm{d}t}\left(S^{n+1}_{i,x} - S^{n}_{i,x}\right), \tag{44}$$

$$u^{(r),n+1/2}_{i,x} = \left(\frac{s_{i,x+1}}{s_{i,x}}\right) u^{(l)}_{i,x+1}. \tag{45}$$

At a terminating branch ($e_i = e_{z,N_z^{\mathrm{cond}}+N^{\mathrm{acin}}-1,k} \in \mathcal{L}$) the final volume-unit has velocity boundary condition $u^{(r)}_{i,p_i-1} = 0$. At a bifurcation, conservation of flux means

$$u^{(r),n+1/2}_{i,p_i-1} = \frac{1}{s_{i,p_i-1}}\left(u^{(l)}_{i_{d1},0} s_{i_{d1},0} + u^{(l),n+1/2}_{i_{d2},0} s_{i_{d2},0}\right) \tag{46}$$

where $e_{i_{d1}}$ and $e_{i_{d2}(i)}$ are the daughter branches of e_i. The no-flow boundary conditions equations (44), (45), and (46) fully define the velocity everywhere on the tree. The final step is to update the

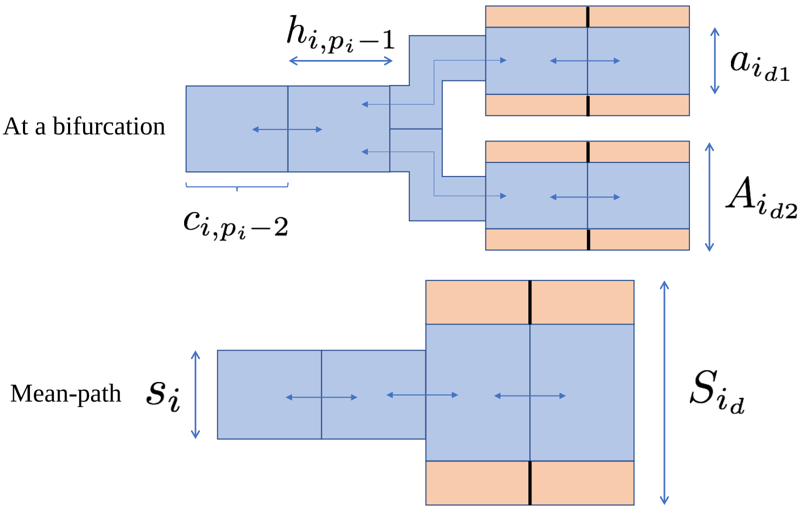

Figure SF4: Top: Sketch of a volumes and fluxes at a branching point between generations $j = N_z^{\text{cond}}$ and $N_z^{\text{cond}} + 1$. The cross-sectional areas of the daughter tubes with indices i_{d1} and i_{d2} are labelled, as well as the volume length and concentration. The z-shaped tubes are for visualisation purposes only, highlighting the flux-splitting. Bottom: The equivalent volumes in a mean-path branch, no flux splitting takes place and both daughters are represented by the same tube with index i_d. The mean-path cross-sections are illustrated.

concentration field using the Crank-Nicholson discretisation of equation (16),

$$\begin{aligned} S_{i,x}^{n+1} c_{i,x}^{n+1} + \frac{\mathrm{d}t}{2h_{i,x}} \left(F_{i,x+1/2}^{(a),n+1} + F_{i,x+1/2}^{(d),n+1} - F_{i,x-1/2}^{(a),n+1} - F_{i,x-1/2}^{(d),n+1} \right) = \\ S_{i,x}^{n} c_{i,x}^{n} - \frac{\mathrm{d}t}{2h_{i,x}} \left(F_{i,x+1/2}^{(a),n} + F_{i,x+1/2}^{(d),n} - F_{i,x-1/2}^{(a),n} - F_{i,x-1/2}^{(d),n} \right) \end{aligned} \tag{47}$$

where the advective and diffusive fluxes are defined respectively as

$$F_{i,x+1/2}^{(a),n} = s_{i,x+1} \left[\max\left(u_{i,x+1}^{(l),n+1/2}, 0 \right) c_{i,x}^{n} + \min\left(u_{i,x+1}^{(l),n+1/2}, 0 \right) c_{i,x+1}^{n} \right] \tag{48}$$

$$F_{i,x+1/2}^{(d),n} = D_{i,x+1/2}^{n+1/2} \left(\frac{c_{i,x+1}^{n} - c_{i,x}^{n}}{h_{i,x} + h_{i,x+1}} \right) \min\left(s_{i,x} \,,\ s_{i,x+1} \right) \tag{49}$$

$$F_{i,x+1/2}^{(a),n+1} = s_{i,x+1} \left[\max\left(u_{i,x+1}^{(l),n+1/2}, 0 \right) c_{i,x}^{n+1} + \min\left(u_{i,x+1}^{(l),n+1/2}, 0 \right) c_{i,x+1}^{n+1} \right] \tag{50}$$

$$F_{i,x+1/2}^{(d),n+1} = D_{i,x+1/2}^{n+1/2} \left(\frac{c_{i,x+1}^{n+1} - c_{i,x}^{n+1}}{h_{i,x} + h_{i,x+1}} \right) \min\left(s_{i,x} \,,\ s_{i,x+1} \right) \tag{51}$$

such that the diffusive flux is determined by the smaller cross-section, and the advective flux is first-order upwinded. The diffusion constant D is

(52)

$$D_{i,x+1/2}^{n+1/2} = \begin{cases} D_0 + C\sqrt{a_i/\pi} \left(u_{i,x}^{(r),n+1/2} + u_{i,x}^{(l),n+1/2} \right) & \forall e_i \in \mathcal{E}_Z^{\text{cond}}, \\ D_0 \left[\phi \left(A_i^n + A_i^{n+1} \right)/2 + (1-\phi) a_i \right]/a_i & \forall e_i \in \mathcal{E}_Z^{\text{acin}} \end{cases} \tag{53}$$

At a terminal volume element ($w_{i,p_i-1} \in e_i$ for any $e_i = e_{z,N_z^{\text{cond}}+N^{\text{acin}}-1,k} \in \mathcal{L}$) the fluxes at the end of the tree are defined as zero $F_{i,p_i-1/2}^{(a)}, F_{i,p_i-1/2}^{(d)} = 0$. Finally, at a bifurcation where the daughter

airways are labelled $e_{i_{d1}}$ and $e_{i_{d2}}$ (see figure SF4) the update equation is

$$\begin{aligned} S^{n+1}_{i,p_i-1}c^{n+1}_{i,p_i-1} + \frac{\mathrm{d}t}{2h_{i,p_i-1}}\left(F^{(a),n+1}_{i_{d1},-1/2} + F^{(d),n+1}_{i_{d1},-1/2} + F^{(a),n+1}_{i_{d2},-1/2} + F^{(d),n+1}_{i_{d1},-1/2} - F^{(a),n+1}_{i,p_i-3/2} - F^{(d),n+1}_{i,p_i-3/2}\right) \\ = S^{n}_{i,p_i-1}c^{n}_{i,p_i-1} - \frac{\mathrm{d}t}{2h_{i,p_i-1}}\left(F^{(a),n}_{i_{d1},-1/2} + F^{(d),n}_{i_{d1},-1/2} + F^{(a),n}_{i_{d2},-1/2} + F^{(d),n}_{i_{d2},-1/2} - F^{(a),n}_{i,p_i-3/2} - F^{(d),n}_{i,p_i-3/2}\right). \end{aligned} \tag{54}$$

Fluxes at the bifurcation are defined consistently with equations (48)-(51). Transport through the mouth dead-space V_{mouth} is represented by a simple piston-like (advection only) transport. We introduce a (zero volume) imaginary node at the top of the tracheal branch e_{trachea} with concentration $c_{\text{trachea},-1}$ equivalent to the concentration at the upper airway). On inhalation, the concentration at the mouth entrance is $c_m(t) = 0$ and $c_{\text{trachea},-1} = c_{\text{trachea},0}$ is determined by advection in the mouth volume. On exhalation, $c_{\text{trachea},-1} = c_{\text{trachea},0}$ and $c_m(t) = 0$ is determined by advection in the mouth volume. The set of equations for $\mathbf{c}^{n+1}$ is a sparse linear system that we solve iteratively in C++ using the Eigen [11] 'BiCGSTAB' routine. To speed up computation we use the forward Euler method as an initial guess for the iterative computation

$$c^{n+1}_{i,x} \approx \frac{1}{S^{n+1}_{i,x}}\left[S^{n}_{i,x}c^{n}_{i,x} - \frac{\mathrm{d}t}{h_{i,x}}\left(F^{(a),n}_{i,x+1/2} + F^{(d),n}_{i,x+1/2} - F^{(a),n}_{i,x-1/2} - F^{(d),n}_{i,x-1/2}\right)\right] \tag{55}$$

and at bifurcations

$$\begin{aligned} c^{n+1}_{i,p_i-1} \approx \frac{1}{S^{n+1}_{i,p_i-1}}\Bigg[S^{n}_{i,p_i-1}c^{n}_{i,p_i-1} - \frac{\mathrm{d}t}{h_{i,p_i-1}}\Big(F^{(a),n}_{i_{d1},-1/2} + F^{(d),n}_{i_{d1},-1/2} \\ + F^{(a),n}_{i_{d2},-1/2} + F^{(d),n}_{i_{d2},-1/2} - F^{(a),n}_{i,p_i-3/2} - F^{(d),n}_{i,p_i-3/2}\Big)\Bigg] \end{aligned} \tag{56}$$

Following the update on the baseline model, we update the linearly perturbed equations on each linear perturbed tree. This involves computing the $O(\epsilon^1)$ changes to all equations, taking into account the difference in tree structure from model M. These equations are then solved in the same manner as the model M ($O(\epsilon^0)$) equations, and depend on the updated $O(\epsilon^0)$ properties. To summarise, at each time step the simulation update is performed as follows

1. Update model M volumes through equation (42).
2. Update model M alveolar volumes through equation (7).
3. Update model M flow speed through incompressibility relations (45)-(46).
4. Update model M concentrations through equations (47) and (54).
5. Update linearly perturbed flows on each perturbed network of model P (see Fig 2 of main text).
6. Update linearly perturbed concentration on each perturbed network of model P (if included).

3 Multiple breath washout test and ventilation heterogeneity

The lung clearance index (LCI) is calculated by $\text{LCI} = V_{\text{exh}}(t_{\text{LCI}})/V^{\text{approx}}_{\text{FRC}}(t_{\text{LCI}})$ where $V_{\text{exh}}(t)$ is the cumulative volume expired during washout. The FRC of the lung $V^{\text{approx}}_{\text{FRC}}(t)$ is itself approximated from the washout curve as

$$V^{\text{approx}}_{\text{FRC}}(t) = \frac{\int_0^t \dot{V}_{\text{exh}}(\tilde{t})c_m(\tilde{t})\mathrm{d}\tilde{t}}{c_m(0) - c_m(t)} \tag{57}$$

where $c_m(t)$ is the concentration measured at the mouth, at $t = 0$ this is equal to the equilibrium concentration at end of wash-in. Throughout we use $V_{\text{FRC}}^{\text{approx}}(t_{\text{LCI}})$ as the MBW determined FRC volume, where t_{LCI} is the time (at end-tidal) when the concentration is below the LCI threshold of $c_m(t) < 0.025c_m(t = 0)$.

The phase-III slopes $Sn_{\text{III}}(N_{TO}(t_i))$ (units L^{-1}) of the individual exhalations are gradients of the final 'phase' (in tidal breathing) of the concentration-volume curve for a given exhalation n, normalised by the average concentration. Following [13] we use the final 50% of exhaled volume on each breath. Additionally, the index S_{cond} is the (fitted) slope of increase in Sn_{III} values versus $N_{TO}(t_i)$ between $N_{TO} = 1.5$-6.

In hyperpolarised inert gas MRI the fractional ventilation is computed as the inert gas dilution rate (correcting for gas depolarisation) in each voxel of imaged lung tissue [14]. Here, we have similarly define fractional ventilation at the scale of the individual acini $\Omega_{z,\alpha}$ for breath number i as

$$FV_{z,\alpha}^{(i)} = \frac{\sum_{j,k} \int_0^{l_{z,j,k}} c_{z,j,k}(x, t_i) A_{z,j,k}(t_i) \mathrm{d}x}{\sum_{z,j,k} \int_0^{l_{z,j,k}} c_{z,j,k}(x, t_{i-1}) A_{z,j,k}(t_{i-1}) \mathrm{d}x} \,\forall \left\{ j, k \mid e_{z,j,k} \in \mathcal{S}_{z, N_z^{\text{cond}}, \alpha} \right\}. \tag{58}$$

The mean fractional ventilation over the whole test is then simply $FV_{z,\alpha} = \sum_{i=0}^{N_{\text{breaths}}} FV_{z,\alpha}^i / N_{\text{breaths}}$ where N_{breaths} is the total number of breaths in the test.

3.1 Numerical modelling of multiple breath washout test

We have simulated a MBW test by applying a fixed flow rate at the mouth assumed to be sinusoidal $q_{\text{mouth}} = \frac{2\pi V_T}{\tau} \sin\left(\frac{2\pi t}{\tau}\right)$ to approximate tidal breathing. This assumption puts a single constraint on the generic ventilation equation (6) (see section 2 for implementation).

The lung model is initialised with a constant (normalised) concentration $c = 1$ of the tracer gas everywhere. In order to accurately determine the LCI, a washout curve is constructed by measuring c_m at each exhalation as the final measured concentration and take the LCI value as the (interpolated) point on the N_{TO}-axis at which this curve crosses $0.025c_m(t = 0)$. Interpolation is used to obtain an LCI that is a continuous variable, sensitive to changes smaller than the turnover fraction V_T/V_{FRC}. In experimental tests variability in breath volumes and test repetition mean that LCI is not restricted to multiples of turnover fraction. Two simulated examples of washout measurements are shown in figure SF5.

The linear sensitivities of the MBW indices $\mathcal{I} \in \{\text{LCI}, S_{\text{cond}}\}$ to perturbations of the mean-path properties are acquired by retaining only linear terms. Following equation (25) the change in these parameters on the whole network $\mathcal{L}$ is approximated as

$$\Delta\mathcal{I} = \sum_{\mathcal{T}_z \in \mathcal{L}} \left\{ \sum_{e_{z,j,k} \in \mathcal{T}_z} \left[\delta\mathcal{I}^{(l_{z,j,k})} \epsilon_{z,j,k}^{(l)} + \delta\mathcal{I}^{(a_{z,j,k})} \epsilon_{z,j,k}^{(a)} \right] + \delta\mathcal{I}^{(K_{z,\alpha})} \sum_{\Omega_{z,\alpha} \in \Omega_z} \epsilon_{z,\alpha}^{(K)} \right\}. \tag{59}$$

The MBW indices $\mathcal{I}$ are measured at the mouth, so the redundancy relations in equation (26) mean that $\delta\mathcal{I}^{(p_{z,j,k})}$ is the same for all perturbations of type p in a given generation j of network $\mathcal{T}_z \in \mathcal{L}$.

The fractional ventilation $FV_{z,\alpha}$ depends on the relative position of perturbations on the network, but following equation (25) can still be reconstructed using the relevant combination of sensitivity functions. For acini $\Omega_{z,\alpha}$ in network $\mathcal{T}_z$ the linear change in FV (using the redundancy relations in

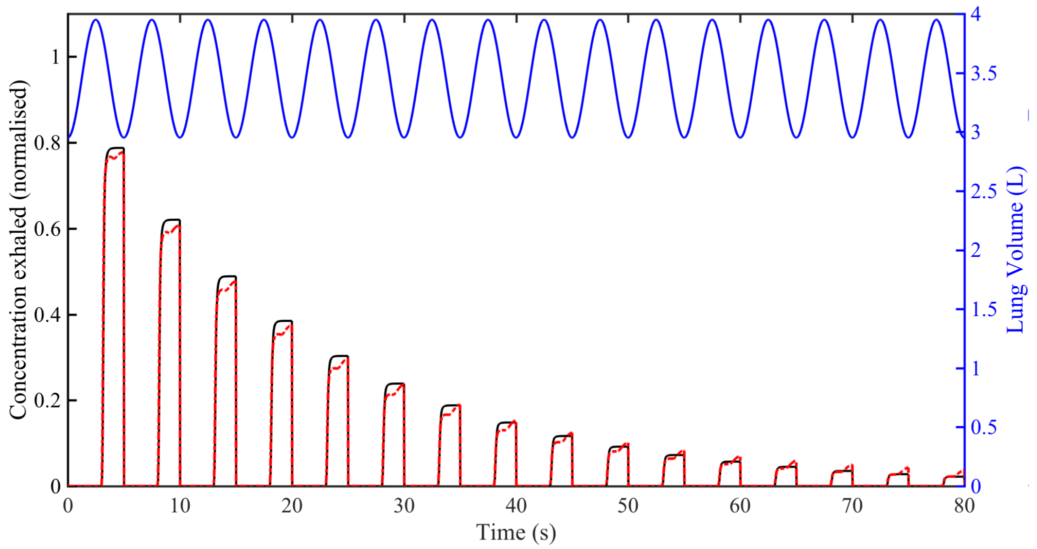

Figure SF5: Washout curves from model M with no constrictions (black solid line) and radius constrictions of the central airways in RM lobe of 78% (red dashed line). The right axis corresponds to the lung volume (blue curve) (not including mouth dead-space) which is taken to be sinusoidal with a tidal volume of 1L.

equation (26)) is

$$\begin{aligned}\Delta FV_{z,\alpha} = &\sum_{\mathcal{T}_{z'} \in \mathcal{L} \setminus \mathcal{T}_z} \left\{ \sum_{e_{j,k} \in \mathcal{T}_{z'}} \left[\delta FV_z^{(l,z',j)} \epsilon_{z',j,k}^{(l)} + \delta FV_z^{(a,z',j)} \epsilon_{z',j,k}^{(a)} \right] + \delta FV_z^{(K,z')} \sum_{\Omega_{z',\tilde{\alpha}} \in \Omega_{z'}} \epsilon_{z',\tilde{\alpha}}^{(K)} \right\} \\ &+ \sum_{e_{j,k} \in \mathcal{T}_z} \left[\delta FV_z^{(l,j,j_{\mathrm{LCA}})} \epsilon_{z,j,k}^{(l)} + \delta FV_z^{(a,j,j_{\mathrm{LCA}})} \epsilon_{z,j,k}^{(a)} \right] + \delta FV_z^{(K,j,j_{\mathrm{LCA}})} \sum_{\Omega_{z,\alpha} \in \Omega_z} \epsilon_{z,\alpha}^{(K)}\end{aligned} \tag{60}$$

The linear sensitivities $\delta FV_z^{(p,z',j)}$ for $p = \{l, a, K\}$ from trees $\mathcal{T}_{z'} \neq \mathcal{T}_z$ depend only on perturbation generation and the tree perturbed, since all perturbations in the same generation of a different tree have the same effect. The second term counts the contributions $\delta FV_z^{(p,j,j_{\mathrm{LCA}})}$ from the within tree $\mathcal{T}_z$, which depend on the perturbation generation j and the LCA generation $j_{\mathrm{LCA}}(z, j, k, N_z^{\mathrm{cond}}, \alpha)$, as defined in equation (27).

4 Model P: Random heterogeneity and summation of linear perturbations

In the linear perturbation limit used in model P, any model parameter is a linear superposition of the linear sensitivity functions weighted by the perturbations (see equation (25)). Hence, if the perturbations are defined as random variables from a multi-variate Gaussian, the variance in any model parameter $g_{z,j,k}$ can be written as the sum of the covariance matrix weighted by the relevant

linear sensitivities:

$$\operatorname{var}(g_{z,j,k}) = \sum_{\mathcal{T}_{z_1},\mathcal{T}_{z_2}\in\mathcal{L}} \left\{ \sum_{e_{j_1,k_1}\in\mathcal{T}_{z_1}, e_{j_2,k_2}\in\mathcal{T}_{z_2}} \left[\sum_{p^{(1)},p^{(2)}\in\{a,l,K\}} \min(1-\delta_{p^{(1)},K}+\delta_{j_1,N^{\text{cond}}_{z_1}},1) \right.\right.$$
$$\left.\left. \min(1-\delta_{p^{(2)},K}+\delta_{j_2,N^{\text{cond}}_{z_2}},1)\operatorname{cov}\left(\epsilon^{(p^{(1)})}_{z_1,j_1,k_1},\epsilon^{(p^{(2)})}_{z_2,j_2,k_2}\right)\delta g^{(p^{(1)}_{z_1,j_1,k_1})}_{z,j,k}\delta g^{(p^{(2)}_{z_2,j_2,k_2})}_{z,j,k}\right]\right\}, \tag{61}$$

where the functions $\min(1-\delta_{p_1,K}+\delta_{j_1,N^{\text{cond}}_{z_1}},1)$ and $\min(1-\delta_{p_2,K}+\delta_{j_2,N^{\text{cond}}_{z_2}},1)$ simply ensure that perturbations to the acinar elasticity occur only at the acinar generation $j = N^{\text{cond}}$. We use this relation used to calculate the variance in MBW indices and FV values for following example cases.

4.1 Independent random heterogeneity

First, we have considered the case where perturbations are independent and normally distributed with zero mean and variance σ_p^2 such that $\epsilon^{(p)}_{z,j,k} = \mathcal{N}(0,\sigma_p^2)$. The covariance function is then $\operatorname{cov}(\epsilon^{(p)}_{z,j,k},\epsilon^{(p')}_{j',k',z'}) = \delta_{j,j'}\delta_{k,k'}\delta_{z,z'}\delta_{p,p'}\sigma_p^2$ where $p\in\{a,l,K\}$ is the perturbation type (area, length or acinar elastance respectively). Using this definition of the covariance and equation (59) (the general expression for the linear sensitivity of the MBW indices), the variance in an MBW index $\mathcal{I}$ is approximated as

$$\operatorname{var}(\mathcal{I}) = \sum_{\mathcal{T}_z\in\mathcal{L}}\left[2^{N^{\text{cond}}_z}\left(\delta\mathcal{I}^{(K,z,N^{\text{cond}}_z)}\sigma_K\right)^2 + \sum_{p\in\{a,l\}}\sum_{j=0}^{N^{\text{cond}}_z+N^{\text{acin}}} 2^j\left(\delta\mathcal{I}^{(p,z,j)}\sigma_p\right)^2\right]. \tag{62}$$

Unlike the MBW indices, the fractional ventilation values are associated with individual acini $\Omega_{z,\alpha}$. Therefore the linear sensitivities depend on the relative position of the defects. Using equation (60)(the general expression for the linear sensitivity of the FV values), the variance in FV (mean value over all breaths) is

$$\begin{aligned}\operatorname{var}(FV_{z,\alpha}) = &\sum_{\mathcal{T}_{z'}\in\mathcal{L}\setminus\mathcal{T}_z}\left[2^{N^{\text{cond}}_{z'}}\left(\delta FV^{(K,z',N^{\text{cond}}_{z'})}_{z,N^{\text{cond}}_z}\sigma_K\right)^2 + \sum_{p\in\{a,l\}}\sum_{j=0}^{N^{\text{cond}}_{z'}+N^{\text{acin}}_{z'}} 2^j\left(\delta FV^{(p,z',j)}_{z,N^{\text{cond}}_z}\sigma_p\right)^2\right]\\
&+\left(\delta FV^{(K,N^{\text{cond}}_z,N^{\text{cond}}_z)}_{z,N^{\text{cond}}_z}\sigma_K\right)^2 + \sum_{j_{\text{LCA}}=0}^{N^{\text{cond}}_z-1} 2^{N^{\text{cond}}_z-j_{\text{LCA}}-1}\left(\delta FV^{(K,N^{\text{cond}}_z,j_{\text{LCA}})}_{z,N^{\text{cond}}_z}\sigma_K\right)^2\\
&+\sum_{p\in\{a,l\}}\left[\sum_{j=1}^{N^{\text{cond}}_z+N^{\text{acin}}_z}\sum_{j_{\text{LCA}}=0}^{\min(j,N^{\text{cond}}_z)-1} 2^{j-j_{\text{LCA}}-1}\left(\delta FV^{(p,j,j_{\text{LCA}})}_{z,N^{\text{cond}}_z}\sigma_p\right)^2\right.\\
&\left.+\sum_{j=0}^{N^{\text{cond}}_z+N^{\text{acin}}_z} 2^{j-\min(j,N^{\text{cond}}_z)}\left(\delta FV^{(p,j,\min(j,N^{\text{cond}}_z))}_{z,N^{\text{cond}}_z}\sigma_p\right)^2\right].\end{aligned} \tag{63}$$

The first line of equation (63) contains the contributions from perturbations in the other SBD regions $z' \neq z$, which depend only on the region and generation (for elasticity perturbations the generation is always $j = N^{\text{cond}}_{z'}$. The second line contains elasticity perturbations in the same lobe, where the first term is the response to a perturbation in acinus $\Omega_{z,\alpha}$ and the second term contains the response to all other elasticity perturbations. This term depends only on their LCA generation, where $j_{\text{LCA}} \equiv j_{\text{LCA}}(z,j,N^{\text{cond}}_z,k,\alpha)$ (defined in (27)) w.r.t. $\Omega_{z,\alpha}$. Similarly, the third line contains contributions from area and length perturbations that are not direct ancestors or descendents of $e_{N^{\text{cond}}_z,\alpha}$, while the final line accounts for area and length perturbations for those branches that are.

4.2 Correlated random heterogeneity

Second, we have considered airway sizes that are inherited from their parent branch such that geometry is correlated with the underlying structure of the lung. This inherited randomness is considered in [15] when modelling particle deposition in a stochastic lung model. In this case, we assume that the perturbations to length and area of branch $e_{j,k}$ are normally distributed with expected value given by the perturbations in their parent branch, such that

$$\begin{aligned} \boldsymbol{\epsilon}_{j,k,z} &= \mathcal{N}\left(\boldsymbol{\epsilon}_{j-1,\lfloor k/2 \rfloor,z}, \boldsymbol{\Sigma}\right), \\ \boldsymbol{\epsilon}_{j,k,z} &= \begin{pmatrix} \epsilon^{(a)}_{j,k,z} \\ \epsilon^{(l)}_{j,k,z} \end{pmatrix}, \; \boldsymbol{\Sigma} = \begin{pmatrix} \sigma_a^2 & \rho_{al}\sigma_a\sigma_l \\ \rho_{al}\sigma_a\sigma_l & \sigma_l^2 \end{pmatrix}. \end{aligned} \tag{64}$$

This results in a covariance between any two pairs of perturbations of $\mathrm{cov}(\boldsymbol{\epsilon}_{j,k,z}, \boldsymbol{\epsilon}_{j',k',z'}) = (j_{\mathrm{LCA}} + 1)\delta_{z,z'}\boldsymbol{\Sigma}$. The coefficient ρ_{al} quantifies the correlation between the area and length deviations of a given airway. To constrain the number of variables, we assume that the variance σ_p^2 and correlation coefficient ρ_{al} do not depend on generation. Using the general equation for the linear sensitivity of the MBW indices (equation (59)), and the expression for the covariance from above, the variance in the MBW indices $\mathcal{I}$ can be reduced to

$$\begin{aligned} \mathrm{var}(\mathcal{I}) = \sum_{\mathcal{T}_z \in \mathcal{L}} \Bigg\{ & 2^{N_z^{\mathrm{cond}}} \left(\delta\mathcal{I}^{(K,z,N_z^{\mathrm{cond}})}\sigma_K\right)^2 + \sum_{p_1,p_2 \in \{a,l\}} \sum_{j_1,j_2=0}^{N_z^{\mathrm{cond}}+N_z^{\mathrm{acin}}} 2^{j_1+j_2}\delta\mathcal{I}^{(p_1,z,j_1)}\delta\mathcal{I}^{(p_2,z,j_2)} \\ & \times \left[2^{-\min(j_1,j_2)}\left(\min(j_1,j_2)+1\right) + \sum_{j_{\mathrm{LCA}}=0}^{\min(j_1,j_2)-1} 2^{-(j_{\mathrm{LCA}}+1)}\left(j_{\mathrm{LCA}}+1\right)\right] \Sigma_{p_1,p_2} \Bigg\}. \end{aligned} \tag{65}$$

In the above, the elastance contribution (first term) remains the same, as this is assumed uncorrelated (both spatially and w.r.t. other variables). The second term sums all contributions from correlated perturbations to generations j_1 and j_2 in the same region (perturbations are independent at inter-regional level). Of these, the first term in the square bracket counts those that are direct ancestors/descendents (*i.e.* $j_{LCA}(j_1,k_1,j_2,k_2) = \min(j_1,j_2)$) and the second counts all other possible relations (where $j_{LCA}(j_1,k_1,j_2,k_2) < \min(j_1,j_2)$).

Computing the variance of FV in the acini $\mathrm{var}(FV_{z,\alpha})$ is more complex, as the contribution depends on the relative position of the two perturbations w.r.t. the SBD region $\mathcal{T}_z$ and w.r.t. each other. This

results in the sum

$$
\begin{aligned}
\mathrm{var}(FV_{z,\alpha}) = \sum_{\mathcal{T}_{z'}\in\mathcal{L}\setminus\mathcal{T}_z} & \left\{ 2^{N_{z'}^{\mathrm{cond}}} \left(\delta FV_{z,N_z^{\mathrm{cond}}}^{(K,z',N_{z'}^{\mathrm{cond}})} \sigma_K \right)^2 + \sum_{p_1,p_2\in\{a,l\}} \Sigma_{p_1,p_2} \sum_{j_1,j_2=0}^{N_{z'}^{\mathrm{cond}}+N_{z'}^{\mathrm{acin}}} 2^{j_1+j_2} \delta FV_z^{(p_1,z',j_1)} \right. \\
& \left. \times \delta FV_z^{(p_2,z',j_2)} \left[2^{-\min(j_1,j_2)} \left(\min(j_1,j_2)+1\right) + \sum_{j'_{\mathrm{LCA}}=0}^{\min(j_1,j_2)-1} 2^{-(j'_{\mathrm{LCA}}+1)} \left(j'_{\mathrm{LCA}}+1\right) \right] \right\} \\
& + \left(\delta FV_{z,N_z^{\mathrm{cond}}}^{(K,N_z^{\mathrm{cond}},N_z^{\mathrm{cond}})} \sigma_K \right)^2 + \sum_{j_{\mathrm{LCA}}=0}^{N_z^{\mathrm{cond}}-1} 2^{N_z^{\mathrm{cond}}-j_{\mathrm{LCA}}-1} \left(\delta FV_{z,N_z^{\mathrm{cond}}}^{(K,N_z^{\mathrm{cond}},j_{\mathrm{LCA}})} \sigma_K \right)^2 \\
& + \sum_{p_1,p_2\in\{a,l\}} \Sigma_{p_1,p_2} \sum_{j_1,j_2=0}^{N_z^{\mathrm{cond}}+N_z^{\mathrm{acin}}} 2^{j_1+j_2} \\
& \times \Bigg\{ 2^{-\min(j_1,N_z^{\mathrm{cond}})-\min(j_2,N_z^{\mathrm{cond}})} \delta FV_z^{(p_1,j_1,\min(j_1,N_z^{\mathrm{cond}}))} \delta FV_z^{(p_2,j_2,\min(j_2,N_z^{\mathrm{cond}}))} \\
& \quad \times \left[2^{-\max(\min(j_1-N_z^{\mathrm{cond}},j_2-N_z^{\mathrm{cond}}),0)} \left(\min(j_1,j_2)+1\right) + \sum_{j'_{\mathrm{LCA}}=N_z^{\mathrm{cond}}}^{\min(j_1,j_2)-1} 2^{-(j'_{\mathrm{LCA}}+1-N_z^{\mathrm{cond}})} \left(j'_{\mathrm{LCA}}+1\right) \right] \\
& \quad + 2^{-\min(j_1,N_z^{\mathrm{cond}})} \left[\sum_{j_{\mathrm{LCA}}^{(2)}=0}^{\min(j_2,N_z^{\mathrm{cond}})-1} 2^{-(j_{\mathrm{LCA}}^{(2)}+1)} \left(\min(j_1,j_{\mathrm{LCA}}^{(2)})+1\right) \delta FV_z^{(p_2,j_2,j_{\mathrm{LCA}}^{(2)})} \right] \\
& \quad + \sum_{j_{\mathrm{LCA}}^{(1)}=0}^{\min(j_1,N_z^{\mathrm{cond}})-1} 2^{-(j_{\mathrm{LCA}}^{(1)}+1)} \delta FV_z^{(p_1,j_1,j_{\mathrm{LCA}}^{(1)})} \\
& \quad \times \left[2^{-\min(j_2,N_z^{\mathrm{cond}})} \delta FV_z^{(p_2,j_2,\min(j_2,N_z^{\mathrm{cond}}))} \left(\min(j_2,j_{\mathrm{LCA}}^{(1)})+1\right) + \sum_{j_{\mathrm{LCA}}^{(2)}=0}^{\min(j_2,N_z^{\mathrm{cond}})-1} \delta FV_z^{(p_2,j_2,j_{\mathrm{LCA}}^{(2)})} \right. \\
& \quad \times \Bigg(\left\{1-\delta_{j_{\mathrm{LCA}}^{(1)},j_{\mathrm{LCA}}^{(2)}}\right\} 2^{-(j_{\mathrm{LCA}}^{(2)}+1)} \left\{\min(j_{\mathrm{LCA}}^{(1)},j_{\mathrm{LCA}}^{(2)})+1\right\} \\
& \quad + \delta_{j_{\mathrm{LCA}}^{(1)},j_{\mathrm{LCA}}^{(2)}} \Bigg\{ 2^{-\min(j_1,j_2)} \left[\min(j_1,j_2)+1\right] \\
& \quad \left.\left. + \sum_{j'_{\mathrm{LCA}}=j_{\mathrm{LCA}}^{(1)}+1}^{\min(j_1,j_2)-1} 2^{-(j'_{\mathrm{LCA}}+1)} \left(j'_{\mathrm{LCA}}+1\right) \Bigg\} \Bigg) \right] \right] \Bigg\}.
\end{aligned}
\tag{66}
$$

The first two lines contains the contributions from other regions ($\mathcal{T}_{z'} \neq \mathcal{T}_z$). Within that sum, the first term is the contribution from the (independent) elastance perturbations and the second sums contributions from pairs of airway perturbations (at generations j_1, j_2), where the first term in the square bracket counts the cases where j_1 and j_2 are direct ancestors/descendants of one another, and the second counts all of the other cases where they have mutual LCA generation $j'_{\mathrm{LCA}} < \min(j_1, j_2)$. The third line contains the terms for elastance perturbations within $\mathcal{T}_z$ with the first term contributing the case where the perturbation is to the acinus $\Omega_{z,\alpha}$ and the second counts all perturbations to other

acini, grouped by their LCA generation $j_{\rm LCA}$ w.r.t $\Omega_{z,\alpha}$.

The remaining lines of equation (66) (fourth line onwards) contain all of the contributions from airway perturbations (at generations j_1, j_2) within $\mathcal{T}_z$. The fifth and sixth lines contain the contributions for when e_{z,j_1,k_1} and e_{z,j_2,k_2} are both direct ancestors or descendants of $e_{z,N_z^{\rm cond},\alpha}$, where the first term in the square bracket counts terms where e_{z,j_1,k_1} and e_{z,j_1,k_1} are direct ancestors/descendants of one another (always true if $j_1 \leq N_z^{\rm cond}$ or $j_2 \leq N_z^{\rm cond}$), while the second term counts the terms where they have LCA generation $j'_{\rm LCA} \geq N_z^{\rm cond}$ (possible only when $j_1 > N_z^{\rm cond}$ and $j_2 > N_z^{\rm cond}$). The seventh line counts the terms where e_{z,j_1,k_1} only is a direct ancestor or descendant of $e_{z,N_z^{\rm cond},\alpha}$. Finally, the eighth line onwards contains all the terms where e_{z,j_1,k_1} is not a direct ancestor or descendant of $e_{z,N_z^{\rm cond},\alpha}$ (it has LCA generation $j^{(1)}_{\rm LCA} < \min(j_1, N_z^{\rm cond})$ w.r.t. $e_{z,N_z^{\rm cond},\alpha}$). Within that the first term in the square bracket contains the contribution for terms where e_{z,j_2,k_2} is a direct ancestor or descendant of $e_{z,N_z^{\rm cond},\alpha}$. Otherwise, e_{z,j_2,k_2} has LCA generation $j^{(2)}_{\rm LCA} < \min(j_2, N_z^{\rm cond})$ w.r.t. $e_{z,N_z^{\rm cond},\alpha}$. In that case there are two further possibilities to consider, if $j^{(1)}_{\rm LCA} \neq j^{(2)}_{\rm LCA}$ then the two perturbations cannot be direct ancestors/descendants of one another and their LCA generation w.r.t. each other is $\min(j^{(1)}_{\rm LCA}, j^{(2)}_{\rm LCA})$ (tenth line), otherwise one has to count the different possible relations between e_{z,j_1,k_1} and e_{z,j_2,k_2} (eleventh and twelfth lines) as before.

4.3 Fractional ventilation distribution

The distribution of Fractional Ventilation in any region is, in the linear limit of model P, a normal distribution $P(FV_{z,\alpha})$ with mean $\bar{FV}_z$ (*i.e.* its value in model M) and variance given by either (63) or (66). It follows that the overall probability density functions plotted in Fig 6 of the main text, which represent the distribution of FV in the whole lung, are simply computed from a weighted sum of the relevant normal distributions

$$P(FV_\alpha) = \frac{\sum_{\mathcal{T}_z \in \mathcal{L}} 2^{N_z^{\rm cond}} P(FV_{z,\alpha})}{\sum_{\mathcal{T}_z \in \mathcal{L}} 2^{N_z^{\rm cond}}}. \tag{67}$$

5 Two-Component Model

We use an analytical two-component model of ventilation to check the validity of the predictions made by model M. In this description, the lung is modelled as consisting of a slow and fast compartment, where the airway resistance of the slow compartment is elevated by constrictions. The full system of equations governing the gas volume of the compartments are

$$r_{\rm UA}\dot{V}_0(t) = P_m - P_0(t) \tag{68}$$

$$\left(r_{\rm slow} + \frac{r_{\rm acin}}{X}\right)\dot{V}_{\rm slow}(t) + K\left(V_{\rm slow}(t) - XV_{\rm A}\right) = P_0(t) - P_{pl}(t) \tag{69}$$

$$\left(r_{\rm fast} + \frac{r_{\rm acin}}{1-X}\right)\dot{V}_{\rm fast}(t) + K\left(V_{\rm fast}(t) - (1-X)V_{\rm A}\right) = P_0(t) - P_{pl}(t) \tag{70}$$

$$\dot{V}_{\rm slow} + \dot{V}_{\rm fast} = \dot{V}_0, \tag{71}$$

where $\dot{V}_{\rm fast}$ and $\dot{V}_{\rm slow}$ are the rate of expansion of the fast and slow lung units respectively, and $\dot{V}_0$ is the volumetric flow rate through the upper-airway and proximal airways, which are taken to be common dead-space. The slow units constitute a fraction X of the total acinar volume V_A at equilibrium. The upper airways have linear resistance $r_{\rm UA}$, and the airways connected directly to the slow and fast units

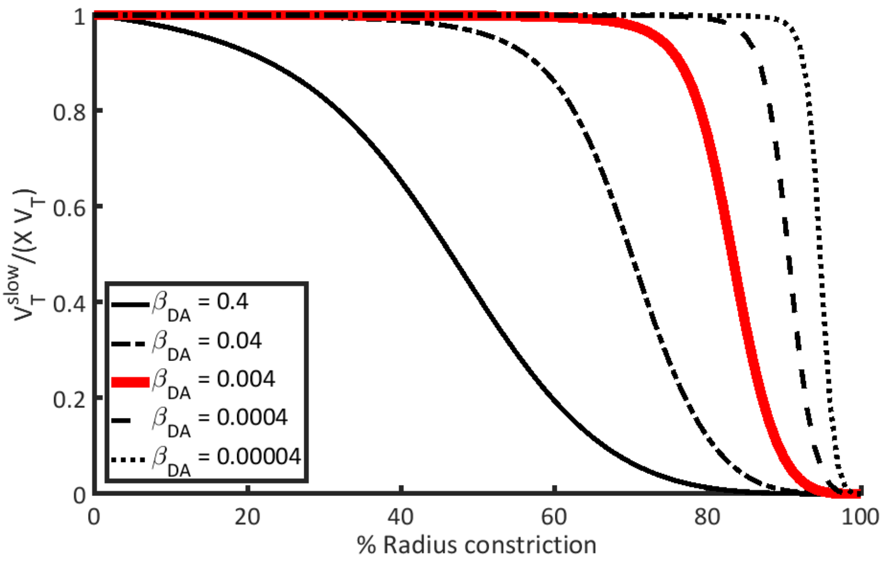

Figure SF6: Plot of V_T^{slow} versus constriction magnitude c scaled by $V_T^{\mathrm{slow}}(c=0) = XV_T$. Five values of β_{DA} are plotted, with the line most closely corresponding to model M ($\beta_{\mathrm{DA}} = 0.004$) highlighted in red and bold. The result depends only weakly with the other parameters which were taken to match model M also ($\beta_{\mathrm{acin}} = 0.05$, $X = 0.1$). The fraction of generations constricted was taken to be $f_c = 1/3$ to approximate model M also.

have resistance r_{slow} and r_{fast} respectively. In order to simulate the effect of localised constrictions we assume

$$r_{\mathrm{fast}} = \frac{r_{\mathrm{DA}}}{1-X}\ , \quad r_{\mathrm{slow}} = \frac{r_{\mathrm{DA}}}{X}\left[1+Z(c)\right]\ , \quad Z(c) \equiv \frac{1}{(1-c)^4} - 1 \tag{72}$$

such that r_{DA} is the baseline resistance of the distal airways and a fraction f_c of the airway generations in the slow region are constricted by factor c of the radius. The effective resistance of all the acini is taken to be r_{acin}, and is assumed homogeneous, as is the elastance K. The pressure P_m is the gas pressure at the mouth, P_0 the pressure at the intersection of the common and independent dead-space, and $P_{pl}(t)$ the applied plural pressure. Subtracting (68) from either equation (69) or (70), eliminates $P_0(t)$. Subtracting the resulting equations eliminates P_{pl} and substituting in equation (71) gives

$$\left\{r_{\mathrm{acin}} + r_{\mathrm{DA}}\left[1+(1-X)Z(c)\right]\right\}\frac{\dot{V}_{\mathrm{slow}}(t)}{X} + \left(r_{\mathrm{DA}} + r_{\mathrm{acin}}\right)\dot{V}_0(t) + K\left[\frac{V_{\mathrm{slow}}(t)}{X} - V_A\right] = 0 \tag{73}$$

Using the same breathing rate $\dot{V}_0 = \frac{V_T\pi}{2\tau}\sin\left(\frac{\pi t}{\tau}\right)$ as in model M. We solve equation (73) for V_{slow} and take the steady state limit ($t \to \infty$) to find

$$\begin{aligned} V_{\mathrm{slow}} = XV_A + \frac{XV_T}{2}\Bigg\{1 - &\frac{\pi\beta_{\mathrm{DA}}(1-X)Z(c)}{1+\pi^2\left[\beta_{\mathrm{acin}} + \beta_{\mathrm{DA}}\left(1+(1-X)Z(c)\right)\right]^2}\sin\left(\frac{\pi t}{\tau}\right) \\ &- \frac{1+\pi^2\left[\beta_{\mathrm{acin}}+\beta_{\mathrm{DA}}\right]\left[\beta_{\mathrm{acin}} + \beta_{\mathrm{DA}}\left(1+(1-X)Z(c)\right)\right]}{1+\pi^2\left[\beta_{\mathrm{acin}} + \beta_{\mathrm{DA}}\left(1+(1-X)Z(c)\right)\right]^2}\cos\left(\frac{\pi t}{\tau}\right)\Bigg\}\ . \end{aligned} \tag{74}$$

From equation (74) we find the difference between the (periodic) minima and maxima of V_{slow} giving the tidal volume into the slow lung unit

$$V_T^{(\text{slow})} = XV_T\sqrt{\frac{1+(\beta_{\text{acin}}+\beta_{\text{cond}})^2}{1+[\beta_{\text{acin}}+\beta_{\text{DA}}(1+(1-X)Z(c))]^2}}. \tag{75}$$

The graph in figure SF6 shows V_T^{slow} versus constriction strength c for various values of β_{DA}. In model M $\beta_{\text{DA}} \approx 0.004$ which can be seen to change very little for constriction magnitude $< 70\%$, and then drops of rapidly to zero by circa 90%, corroborating the model M predictions presented in the main text (*c.f.* the measured FRC in Figs 4(e,f) for example).

References

[1] Horsfield K, Dart G, Olson DE, Filley GF, Cumming G. Models of the human bronchial tree. J Appl Physiol. 1971;31(2):207–217. doi:10.1152/jappl.1971.31.2.207

[2] Pedley TJ, Schroter RC, Sudlow MF. The prediction of pressure drop and variation of resistance within the human bronchial airways. Respir Physiol. 1970;9(3):387–405. doi:10.1016/0034-5687(70)90094-0.

[3] Maury B. The Respiratory System in Equations. Milano: Springer Milan; 2013. Available from: `http://link.springer.com/10.1007/978-88-470-5214-7`.

[4] Dutrieue B, Vanholsbeeck F, Verbanck S, Paiva M. A human acinar structure for simulation of realistic alveolar plateau slopes. J Appl Physiol. 2000;89(5):1859–67. doi:10.1152/jappl.2000.89.5.1859

[5] Henry FS, Llapur CJ, Tsuda A, Tepper RS. Numerical Modelling and Analysis of Peripheral Airway Asymmetry and Ventilation in the Human Adult Lung. J Biomech Eng. 2012;134(6):061001. doi:10.1115/1.4006809.

[6] Tsuda A, Henry FS, Butler JP. Gas and aerosol mixing in the acinus. Respir Physiol Neurobiol. 2008;163(1-3):139–149. doi:10.1016/j.resp.2008.02.010.

[7] Weibel ER, Sapoval B, Filoche M. Design of peripheral airways for efficient gas exchange. Respir Physiol Neurobiol. 2005;148(1-2 SPEC. ISS.):3–21. doi:10.1016/j.resp.2005.03.005.

[8] Scherer PW, Shendalman LH, Greene NM, Bouhuys A. Measurement of axial diffusivities in a model of the bronchial airways. J Appl Physiol. 1975;38(4):719–23. doi:10.1152/jappl.1975.38.4.719

[9] Weibel ER. Morphometry of the Human Lung. 1st ed. Springer-Verlag Berlin Heidelberg; 1963.

[10] Haefeli-Bleuer B, Weibel ER. Morphometry of the human pulmonary acinus. Anat. Rec. 1988;220(4):401–414. doi:10.1002/ar.1092200410.

[11] Guennebaud G, Jacob B, Others. Eigen; 2010. Available from: `http://eigen.tuxfamily.org`.

[12] Whitfield CA. PULMsim v1.1; 2018. doi:10.5281/zenodo.1251974.

[13] Robinson PD, Latzin P, Verbanck S, Hall GL, Horsley A, Gappa M, et al. Consensus statement for inert gas washout measurement using multiple- and single- breath tests. Eur Respir J. 2013;41(3):507–522. doi:10.1183/09031936.00069712.

[14] Horn FC, Deppe MH, Marshall H, Parra-Robles J, Wild JM. Quantification of regional fractional ventilation in human subjects by measurement of hyperpolarized 3He washout with 2D and 3D MRI. J Appl Physiol. 2014;116(2):129–139. doi:10.1152/japplphysiol.00378.2013.

[15] Koblinger L, Hofmann W. Aerosol deposition calculations with a stochastic lung model. Acta Phys Hungarica. 1986;59(1):31–34. doi:10.1007/BF03055180.
